# Host-parasite interactions between Acrididae (Orthoptera: Caelifera) and Parasitengona (Acari: Trombidiformes) in the southwestern Zagros Mountains, Iran

**DOI:** 10.1038/s41598-026-56417-5

**Published:** 2026-06-14

**Authors:** Najmeh Kiany, Marjan Seiedy, Masoud Hakimitabar, Mohsen Kiany, Martin Husemann

**Affiliations:** 1 School of Biology and Center of Excellence in Phylogeny of Living Organisms, College of Science, University of Tehran, Tehran, Iran; 2 Department of Horticulture and Plant Protection, College of Agriculture, Shahrood University of Technology, Shahrood, Iran; 3 Department of Biology, College of Sciences, Shiraz University, Shiraz, Iran; 4 Staatliches Museum für Naturkunde Karlsruhe, Karlsruhe, Germany

**Keywords:** Mites, Attachment sites, Ectoparasites, Grasshoppers, Host range, Host specificity, Ecology, Ecology, Zoology

## Abstract

Parasitengona mites (Acari: Trombidiformes) are common parasites of Orthoptera. Yet, we have limited knowledge on host-parasite interactions between these groups. Hence, in this study we investigate the interactions between these mites and their Acrididae hosts in the southwestern Zagros Mountains (Iran). Sampling was conducted at 22 stations, four times across two consecutive years (2020–2021). A total of 5,344 mite larvae were collected from 2,370 grasshoppers, representing 48 species, of which 1,137 individuals (48%) were parasitized. The mean number of mites per infested individual was 4.7; the maximum infestation observed was 103 *Eutrombidium* cf. *trigonum* larvae on *Calliptamus barbarus*. The most abundant mite species was *E. sorbasiensis* (4,282 larvae; 80%). Parasitism was sex-biased, with females more frequently infested than males (52% vs. 43%), likely due to larger body size. Mites exhibited preferences for specific attachment sites on their hosts, varying by mite species and host grooming behavior. Bipartite network analysis revealed a moderate degree of host specialization (H2′ = 0.38), and individual specialization indices (d′) indicated that most mite species were generalists. These findings suggest that while mites exploit a wide host range, infestation pressure is unevenly distributed and shaped by host traits, attachment-site preferences, and habitat quality, highlighting the importance of ecological and evolutionary factors in shaping host-parasite interactions.

**Supplementary Information:**

The online version contains supplementary material available at https://doi.org/10.1038/s41598-026-56417-5.

## Introduction

Currently, because of human impact on the environment and the loss of many natural grasslands, grasshoppers have become one of the more vulnerable insect groups^1^. With them, their associated species are of similar or even higher threat; a range of different parasites infest these insects, among them parasitengone mites^2, 3^. The relationships between insects and mites, both from ecological and evolutionary perspectives, have been examined in numerous articles^4^, yet, much remains to be studied.

One common group of parasites of Orthoptera and many other insects are the parasitengone mites. As a group within the suborder Prostigmata, the cohort Parasitengona encompasses more than 11,000 described species (ca. 5000 terrestrial and ca. 6000 aquatic)^5^. Most Parasitengona larvae live on the bodies of arthropods. Host recognition and attachment occur through direct contact between the mite larva and the host^6^.

Despite numerous published papers, most studies focus solely on the parasite and fail to identify host species. Moreover, host-parasite relationships are often examined only at higher taxonomic levels^7, 8^. Although several studies have reviewed the host-parasite associations between arthropods and their ectoparasitic mites^9, 10, 6, 11, 12, 13, 14, 7, 15, 16^, a comprehensive, species-level understanding of these interactions remains limited. Orthoptera are the main hosts for Parasitengona, especially species in the family Acrididae^6, 17, 18^. These mites can negatively affect their hosts’ fecundity and survival^19, 20^, Furthermore, infestations of flying hosts may play a critical role in the dispersal of mite populations^6^. There is a long evolutionary history of insect infestations by mites, and erythraeid mites are particularly abundant in Middle Eocene Baltic amber and Early Cretaceous Spanish amber, compared with Microthrombidiidae, with dipterans serving as their primary hosts during that period^21, 22, 23^.

In Iran, both Parasitengona and the grasshopper fauna have received growing research attention^24, 25, 26, 27, 28, 29, 30, 31, 32, 33^. However, only one study has quantitatively and qualitatively evaluated mite parasitism in this region^34^. Iran represents a unique biogeographic transition zone, where three major regions – the Palearctic, the Saharo-Arabian and the Oriental regions – converge; this contributes to its exceptional biodiversity and high level of endemism^35, 36^. The Zagros Mountains represent the northeastern edge of the Arabian plate^37^, located inside the Irano-Anatolian hotspot and along the western side of the Iranian Plateau^35, 38^. The area’s complex topography and high elevation make it one of the main centers of endemism in Iran^39^ and therefore a region of particular ecological importance.

Thus, the present study aims to investigate the Parasitengona mite species associated with Acrididae hosts in the southeastern part of the Zagros Mountains. This paper concludes a series of studies that have explored this host-parasite relationship in the region^40, 41, 42, 43^.

## Materials and methods

### Sampling

**Fig. 1:**
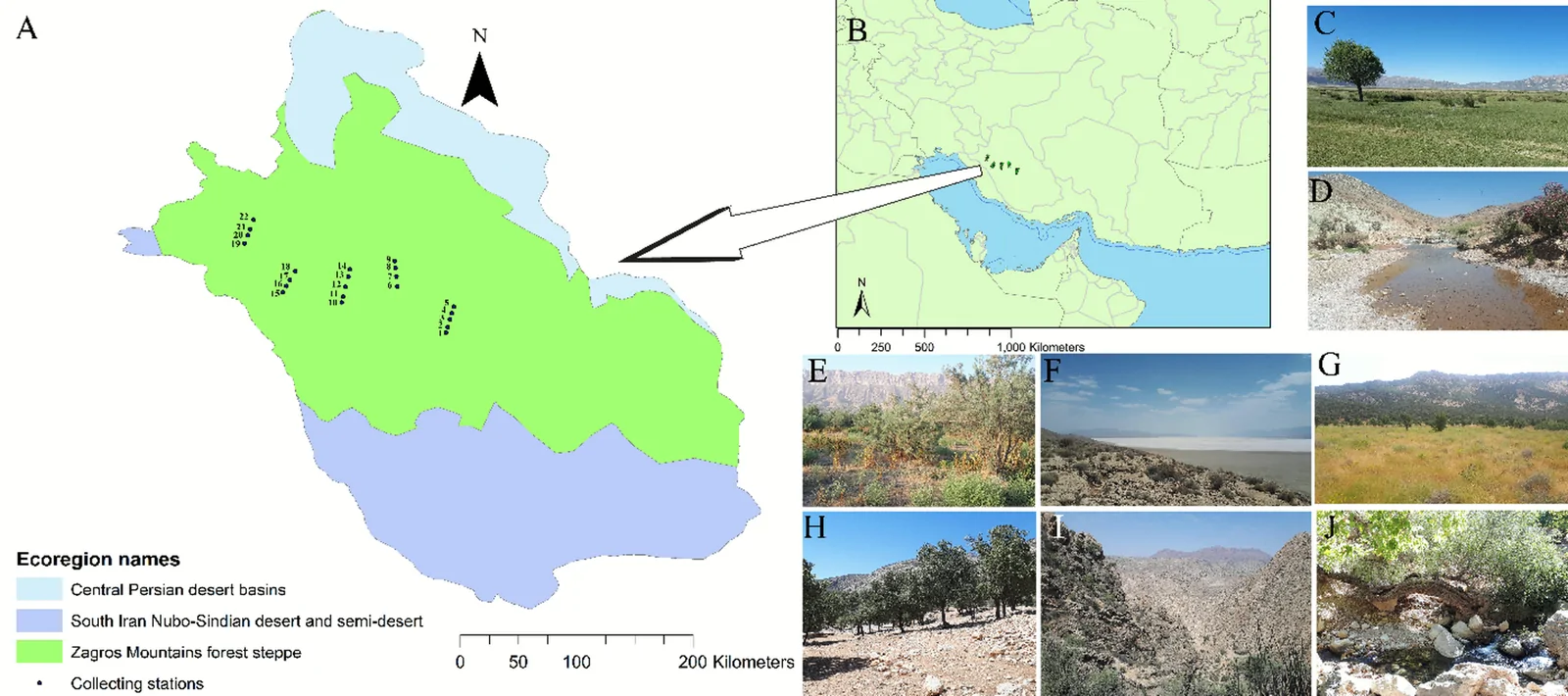
Specimens collecting sites, (**A**,**B**) sampling station located in Zagros mountains forest steppe in Fars Province, Iran. (1) Shoorijeh; (2) Roknabad; (3) Sarvestan; (4) Tangeghanimi; (5) Kharameh; (6) Kaftarak; (7) Cheshmehchenarak; (8) Bardej; (9) Bamu; (10) Darengoon; 11. Ghareaghaj; 12. Siakh; 13. Amirkabir; 14. Derak; 15. Parishan; 16. Dashtebarm; 17. Arjan protected area; 18. Arjan lagoon; 19. Meidanak; 20. Galgun; 21. Khomezar; 22. Bovan ; C-J, different collecting stations presenting Zagros vegetation cover, Ecoregions and provincial boundaries of Iran. Base map: DIVA-GIS; Ecoregions: Dinerstein et al.^44^; photographs taken by the first author.

Grasshoppers and their ectoparasitic mites were collected using insect nets during a two-year faunistic survey (2020 and 2021) in the southeastern part of the Zagros Mountains, Fars Province, Iran (Fig. 1A).

The study area, where both mite larvae and their grasshopper hosts were collected, spans latitudes 29°22’50.71"N–30°03’11.23"N and longitudes 51°35’12.64"E–53°08’37.72"E (Fig. 1B).

The region was divided into five transects, each approximately 22 to 28 km in length. The distance between adjacent transects ranged from 36 to 42 km. A total of 22 stations (Fig. 1A-J) were sampled four times (Table 1).

**Table 1:** Sampling dates at each station and transect.

Stations number	Stations	Sampling date	Sampling number-transect number
1	Shoorijeh	10.6.2020	1–1
2	Roknabad	10.6.2020	1–1
3	Sarvestan	10.6.2020	1–1
4	Tangeghanimi	10.6.2020	1–1
5	Kharameh	10.6.2020	1–1
6	Kaftarak	18.5.2020	1–2
7	Cheshmehchenarak	13.7.2020	1–2
8	Bardej	19.6.2020	1–2
9	Bamu	19.6.2020	1–2
10	Darengoon	30.6.2020	1–3
11	Ghareaghaj	30.6.2020	1–3
12	Siakh	30.6.2020	1–3
13	Amirkabir	27.5.2020	1–3
14	Derak	5.7.2020	1–3
15	Parishan	3.6.2020	1–4
16	Dashtebarm	3.6.2020	1–4
17	Arjan protected area	3.6.2020	1–4
18	Arjan lagoon	3.6.2020	1–4
19	Meidanak	4.6.2020	1–5
20	Galgun	4.6.2020	1–5
21	Khomezar	4.6.2020	1–5
22	Bovan	4.6.2020	1–5
1	Shoorijeh	3.9.2020	2 − 1
2	Roknabad	3.9.2020	2 − 1
3	Sarvestan	3.9.2020	2 − 1
4	Tangeghanimi	3.9.2020	2 − 1
5	Kharameh	3.9.2020	2 − 1
6	Kaftarak	27.7.2020	2–2
7	Cheshmehchenarak	28.9.2020	2–2
8	Bardej	10.9.2020	2–2
9	Bamu	10.9.2020	2–2
10	Darengoon	20.9.2020	2–3
11	Ghareaghaj	20.9.2020	2–3
12	Siakh	20.9.2020	2–3
13	Amirkabir	9.8.2020	2–3
14	Derak	26.9.2020	2–3
15	Parishan	18.8.2020	2–4
16	Dashtebarm	18.8.2020	2–4
17	Arjan protected area	18.8.2020	2–4
18	Arjan lagoon	18.8.2020	2–4
19	Meidanak	19.8.2020	2–5
20	Galgun	19.8.2020	2–5
21	Khomezar	19.8.2020	2–5
22	Bovan	19.8.2020	2–5
1	Shoorijeh	11.6.2021	3 − 1
2	Roknabad	11.6.2021	3 − 1
3	Sarvestan	11.6.2021	3 − 1
4	Tangeghanimi	11.6.2021	3 − 1
5	Kharameh	11.6.2021	3 − 1
6	Kaftarak	19.5.2021	3 − 2
7	Cheshmehchenarak	14.7.2021	3 − 2
8	Bardej	20.6.2021	3 − 2
9	Bamu	20.6.2021	3 − 2
10	Darengoon	1.7.2021	3–3
11	Ghareaghaj	1.7.2021	3–3
12	Siakh	1.7.2021	3–3
13	Amirkabir	28.5.2021	3–3
14	Derak	29.9.2021	3–3
15	Parishan	4.6.2021	3–4
16	Dashtebarm	4.6.2021	3–4
17	Arjan protected area	4.6.2021	3–4
18	Arjan lagoon	4.6.2021	3–4
19	Meidanak	5.6.2021	3–5
20	Galgun	5.6.2021	3–5
21	Khomezar	5.6.2021	3–5
22	Bovan	5.6.2021	3–5
1	Shoorijeh	4.9.2021	4 − 1
2	Roknabad	4.9.2021	4 − 1
3	Sarvestan	4.9.2021	4 − 1
4	Tangeghanimi	4.9.2021	4 − 1
5	Kharameh	4.9.2021	4 − 1
6	Kaftarak	28.7.2021	4 − 2
7	Cheshmehchenarak	29.9.2021	4 − 2
8	Bardej	11.9.2021	4 − 2
9	Bamu	11.9.2021	4 − 2
10	Darengoon	11.9.2021	4 − 3
11	Ghareaghaj	11.9.2021	4 − 3
12	Siakh	11.9.2021	4 − 3
13	Amirkabir	10.8.2021	4 − 3
14	Derak	27.9.2021	4 − 3
15	Parishan	19.8.2021	4–4
16	Dashtebarm	19.8.2021	4–4
17	Arjan protected area	19.8.2021	4–4
18	Arjan lagoon	19.8.2021	4–4
19	Meidanak	20.8.2021	4–5
20	Galgun	20.8.2021	4–5
21	Khomezar	20.8.2021	4–5
22	Bovan	20.8.2021	4–5

Station selection was primarily based on elevation; the first station in each transect was located at the lowest elevation, while the last station was at the highest. Vegetation diversity was also considered, with each transect subdivided into four to five stations. Several limiting factors influenced station selection and spacing, including access restrictions due to the COVID-19 pandemic, protected areas, military zones, agricultural lands and urban or rural settlements. Consequently, the distance between stations varied from 3.5 to 7.8 km.

Sampling was conducted using a time-based method^45, 1^. Each session lasted two hours. Collectors (Najmeh Kiany and Mohsen Kiany) walked along a straight transect line at a constant speed, maintaining a 2-meter distance between them. Each observer sampled a 1-meter zone on their sides of the transect. After one hour (approximately 2,500 m), the return path was offset by two meters to the right, resulting in a total transect width of eight meters. Thus, the actual sampled area at each station was approximately 20,000 m^2^ (2 ha).

Each grasshopper sample was given a unique number to associate it with its mites, then mounted and stored in an insect box. Identification of hosts and their parasites was based on available keys (Beĭ-Bienko and Mischchenko^46, 2, 47, 48, 49, 50, 51, 52, 53, 54, 55, 56, 57, 58^.

Mites collected from each individual host were detached and transferred to separate containers filled with 75% ethanol. They were then cleared in Nesbitt’s fluid or 100% lactic acid and mounted on microscope slides using Faure’s medium^59, 60^.

Ecoregion classifications were derived from Dinerstein et al.^44^, as well as from datasets accessed via https://ecoregions.appspot.com and https://www.naturalearthdata.com (the latter providing free vector and raster map data). The base map and provincial boundaries were obtained from https://diva-gis.org/data.html. The final map was compiled and subsequently modified in ArcMap within ESRI ArcGIS Desktop version 10.8.2 to align with the most current administrative divisions.

### Comparison of infestation across altitudes and habitats

To compare mite infestation rates across elevations, four altitudinal ranges were defined: (1) 800–1200 m; (2) 1200–1600 m; (3) 1600–2000 m; 4) > 2000 m. For habitat comparison based on vegetation, the sampling stations were grouped into five general categories: (1) Mountainous; (2) Wetlands and riparian zones; (3) Forest steppe; (4) Foothills; (5) Plain. Group comparisons were conducted using the Kruskal-Wallis test, followed by Dunn’s pairwise test. Significant results (p-value < 0.05) are reported in Tables 2 and 3 and Supplementary Table 1.

**Table 2:** Significant associations between mite species and altitude ranges across different grasshopper hosts.

Mite species	Grasshopper species	Comparison	Z	*P*.adj
*Abalakeus lorestanicus* Saboori & Lachinani, 2003^61^	*Oedipoda kurda* Descamp, 1967^62^	1200–1600 m − 1600–2000 m	2.697883	0.041869
		1200–1600 m − 800–1200 m	2.918206	0.021123
	*Oedipoda miniata* (Pallas, 1771)^63^	> 2000 m − 1600-2000 m	− 2.94642	0.019288
	*Sphingoderus carinatus *(Saussure, 1888)^64^	> 2000 m − 1600-2000 m	− 3.53332	0.002462
		1200–1600 m − 1600–2000 m	− 3.80976	8.35E-04
		1600–2000 m − 800–1200 m	2.852706	0.026009
	*Sphingonotus* (*Sphingonotus*) *pilosus* Saussure, 1884^65^	1200–1600 m − 1600–2000 m	3.266614	0.006531
		1200–1600 m − 800–1200 m	2.944486	0.019409
*Charletonia ahwazensis* Haitlinger & Saboori, 2007^66^	*Heteracris pterosticha *(Fischer von Waldheim, 1833)^67^	> 2000 m − 1200-1600 m	− 3.74166	5.48E-04
		1200–1600 m − 1600–2000 m	5.192587	6.22E-07
*Charletonia krendowskyi* (Feider, 1954)^68^	*Acrotylus insubricus *(Scopoli, 1786)^69^	> 2000 m − 1600-2000 m	2.810371	0.029691
*Eutrombidium sorbasiensis* Mayoral & Barranco, 2004^70^	*Acrotylus insubricus *(Scopoli, 1786)^69^	> 2000 m − 1600-2000 m	− 3.57827	0.002075
		1200–1600 m − 1600–2000 m	− 2.91313	0.02147
	*Calliptamus barbarus* (Costa, 1836)^71^	> 2000 m − 1600-2000 m	− 3.08019	0.012412
		1200–1600 m − 1600–2000 m	− 4.32571	9.12E-05
	*Chorthippus* sp.	> 2000 m − 1200-1600 m	− 3.56183	0.001105
	*Notostaurus anatolicus* (Krauss, 1896)	> 2000 m − 800-1200 m	2.744083	0.036408
	*Notostaurus* sp.	> 2000 m − 1600-2000 m	2.053726	0.040002
	*Oedipoda miniata* (Pallas, 1771)^63^	> 2000 m − 800-1200 m	− 3.63176	0.001689
		1200–1600 m − 800–1200 m	− 3.95972	4.50E-04
		1600–2000 m − 800–1200 m	− 5.43024	3.38E-07
	*Sphingoderus carinatus* (Saussure, 1888)^64^	> 2000 m − 1200-1600 m	2.921747	0.020884
		1200–1600 m − 800–1200 m	− 2.96466	0.018181
	*Sphingonotus* (*Sphingonotus*) *pilosus* Saussure, 1884^65^	1200–1600 m − 800–1200 m	− 2.52655	0.069114
	*Sphingonotus* (*Sphingonotus*) *rubescens* (Walker, 1870)^72^	1200–1600 m − 800–1200 m	− 2.73958	0.036911
	*Sphingonotus* (*Sphingonotus*) *theodori* Uvarov, 1923	1200–1600 m − 800–1200 m	− 5.86623	2.67E-08
		1600–2000 m − 800–1200 m	− 3.9851	4.05E-04
	*Sphodromerus luteipes* Uvarov, 1933 ^73^	> 2000 m − 1200-1600 m	3.37569	0.004418
*Eutrombidium cf. trigonum* (Hermann 1804)^74^	*Acrida oxycephala* (Pallas, 1771)^63^	> 2000 m − 1600-2000 m	2	0.0455
	*Calliptamus barbarus* (Costa, 1836)^71^	> 2000 m − 1200-1600 m	5.889317	2.33E-08
		> 2000 m − 1600-2000 m	6.731304	1.01E-10
		> 2000 m − 800-1200 m	4.395191	6.64E-05
	*Chorthippus* (*Chorthippus*) *dorsatus* (Zetterstedt, 1821)^75^	> 2000 m − 1600-2000 m	2.011989	0.044221
	*Oedipoda miniata* (Pallas, 1771)^63^	> 2000 m − 1600-2000 m	3.281005	0.006206
	*Truxalis mesopotamica* Dirsh, 1950^76^	> 2000 m − 1200-1600 m	3.464102	0.001596
		> 2000 m − 1600-2000 m	4.107919	1.20E-04
*Leptus* (*Leptus*) *darvishi* Saboori, Hakimitabar & Khademi, 2018	*Notostaurus anatolicus* (Krauss, 1896)	> 2000 m − 800-1200 m	− 3.01279	0.015532

**Table 3:** Significant habitat preferences of mite species across different grasshopper hosts.

Mite species	Host	Habitats comparison	Z	Adj.Sig.
*Eutrombidium sorbasiensis* Mayoral & Barranco, 2004^70^	*Notostaurus anatolicus* (Krauss, 1896)	Mountain – wetland and riparian	3.89	9E−04
	*Oedipoda miniata* (Pallas, 1771)^63^	Forest steppe – mountain	5.1	5.50E−06
		Forest steppe – plain	2.88	0.04
		Forest steppe – wetland and riparian	6.8	1.00E−10
	*Pyrgodera armata* (Fischer von Waldheim, 1820)	Foothill – forest steppe	2.43	0.015
	*Sphingoderus angustus* Descamps, 1967^62^	Foothill – forest steppe	− 2.66	0.024
		Forest steppe – plain	2.87	0.012
	*Sphingoderus carinatus* (Saussure, 1888)^64^	Foothill – plain	3.11	0.019
		Forest steppe – mountain	3.32	0.0091
		Forest steppe – plain	4.4	0.00011
		Forest steppe – wetland and riparian	3.41	0.0065
	*Sphingonotus (Sphingonotus) rubescens* (Walker, 1870)^72^	Forest steppe – plain	2.92	0.035
	*Sphingonotus* (*Sphingonotus*) *theodori* Uvarov, 1923	Foothill – forest steppe	− 4.04	0.00054
		Forest steppe – mountain	3.57	0.0036
		Forest steppe – wetland and riparian	5.36	8.20E−07
*Eutrombidium* cf. *trigonum * (Hermann 1804)^74^	*Calliptamus barbarus* (Costa, 1836)^71^	Foothill – wetland and riparian	5.41	6.30E−07
		Forest steppe – wetland and riparian	− 4.14	0.00035
		Mountain – wetland and riparian	− 5.65	1.60E−07
	*Sphingoderus carinatus* (Saussure, 1888)^64^	Foothill – wetland and riparian	− 3.05	0.023
		Plain– wetland and riparian	− 3.16	0.016
*Leptus mallahae* Kiany, Seiedy, & Hakimitabar, 2024	*Acrotylus insubricus* (Scopoli, 1786)^69^	Forest steppe – plain	− 3.52	0.0044
		Mountain – plain	− 3.9	0.00096
		Plain– wetland and riparian	3.95	7.90E−05
*Tafreshichyzeria hessabyi* Saboori and Lazarboni, 2007^56^	*Notostaurus anatolicus* (Krauss, 1896)	Forest steppe – wetland and riparian	2.79	0.032

### Statistical analyses

All statistical analyses were performed using IBM SPSS Statistics for Windows, Version 27.0 (IBM Corp., Armonk, NY, USA) and R software version 4.5 (R Core Team, Auckland, New Zealand). Bipartite networks and graphical visualizations were generated in R using the packages *bipartite* (v. 2.21^77^) and *ggplot2* (v. 3.5.2^78^), respectively.

First, data was entered into SPSS and tested for normality. As the data was not normally distributed, non-parametric tests were used. In order to compare two groups, the Chi-square and Mann–Whitney U tests were applied. For comparisons involving more than two groups, the Kruskal-Wallis test followed by Dunn’s pairwise test was used. A p-value below 0.05 was considered statistically significant.

To quantify host-parasite interaction patterns, the *bipartite* package in R was used to calculate both network- and species-level specialization indices^77^; specifically: H₂′ was used to measure overall network-level specialization; d′ values were calculated for each parasite species, indicating their degree of host specificity (ranging from 0 = generalist to 1 = complete specialist). Generality (mean number of host species per parasite) and vulnerability (mean number of parasite species per host) were calculated, treating parasites as the higher trophic level.

**Fig. 2:**
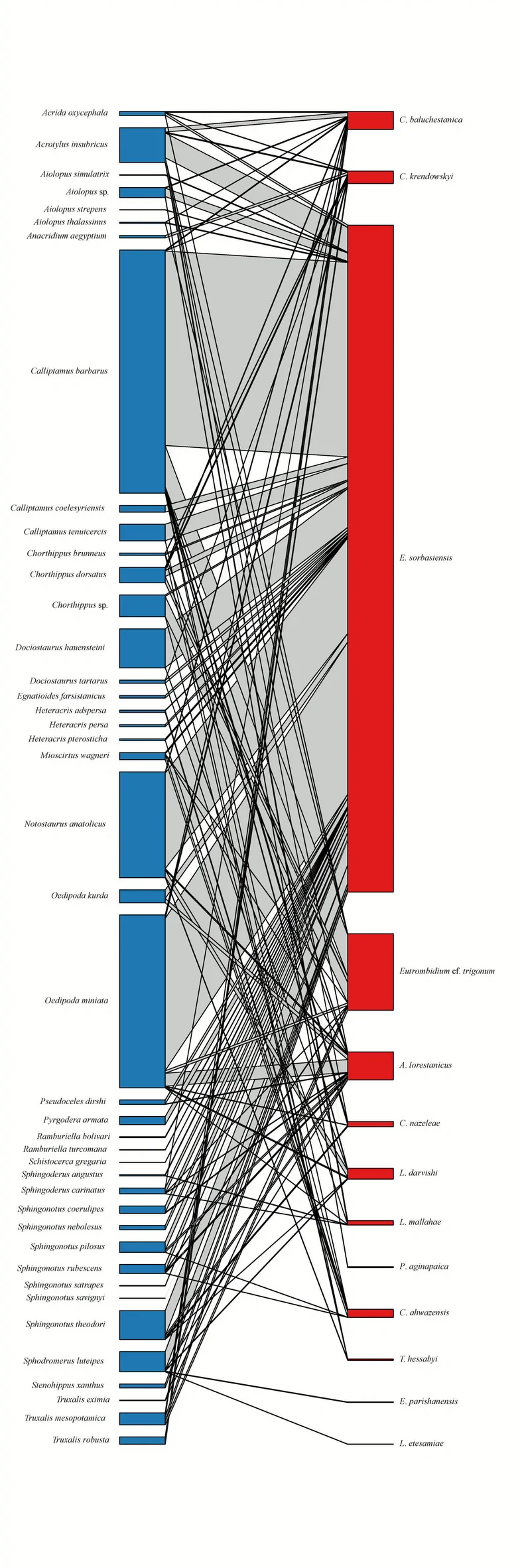
Bipartite network graph showing interactions between mite species (right) and their grasshopper hosts (left). Hosts and parasites are represented by blue and red rectangles, respectively. Links represent observed host–parasite interactions, and link width is proportional to the number of mite individuals recorded on each host species (interaction strength).

Host-parasite interactions were analyzed using bipartite network analysis. The interaction dataset considered grasshopper species, mite species, and the number of mite individuals recorded for each host-parasite association. These abundance values were used as interaction weights to construct the host-parasite network. In the graphical representation (Fig. 2), hosts and parasites were displayed as separate sets of nodes, and the width of each link was proportional to the number of mite individuals observed for a given host–parasite interaction (interaction strength).

To assess community-wide specialization, connectivity (C) was defined as the proportion of realized interactions relative to the total possible interactions in the network, ranging from 0 (no interactions) to 1 (all species connected)^79, 80^.

The prevalence (infestation rate or the percentage of hosts carrying each group of ectoparasite species), intensity (number of parasites on an individual host), mean intensity (total number of parasites divided by the number of parasitized hosts) (Table 4) and preference of the attachment sites were determined (Supplementary Table 1).

**Table 4:** Recorded grasshopper host species and values of parasitological indices of Parasitengona mites collected from the Zagros Mountains: abundance and mean intensity of mites in their grasshopper hosts.

Grasshopper species	Abundance of grasshoppers	Mite prevalence	Total number of mites	Mean mite intensity (± SE)	Intensity	Mean abundance
*Acrida oxycephala *(Pallas, 1771)^63^	♂ 7	71.43%	15	3.00±(1.05)	1–7	2.14±(0.91)
	♀ 3	100.00%	11	3.67±(0.67)	3–5	3.67±(0.67)
	Σ 10	80.00%	26	3.25±(0.67)	1–7	2.60±(0.69)
*Acrotylus insubricus* (Scopoli, 1786)^69^	♂ 77	46.75%	103	2.86±(0.36)	1–10	1.34±(0.24)
	♀ 55	41.82%	115	5.00±(1.18)	1–23	2.09±(0.59)
	Σ 132	44.70%	218	3.69±(0.52)	1–23	1.65±(0.28)
*Acrotylus* spp.	2 immatures	50.00%	4	4	4	2.00±(2.00)
*Aiolopus simulatrix* (Walker, 1870)^72^	♂ 35	0.00%	0	–	–	0
	♀ 4	50.00%	4	–	2	1.00±(0.58)
	Σ 4	50.00%	4	3	2	1.00±(0.58)
*Aiolopus* spp.	♂ 35	14.29%	11	2.20±(0.97)	1–6	0.31±(0.18)
	♀ 28	28.57%	52	6.50±(3.83)	1–33	1.86±(1.18)
	Σ 63	20.63%	63	4.85±(2.40)	1–33	1.00±(0.54)
*Aiolopus strepens* (Latreille, 1804)	♂ 1	0.00%	0	–	–	0
	♀ 1	100.00%	2	2	2	2
	Σ 2	50.00%	2	2	2	1.00±(1.00)
*Aiolopus thalassinus* (Fabricius, 1781)	♂ 10	30.00%	3	1	1	0.30±(0.15)
	♀ 10	30.00%	4	1.33±(0.33)	1–2	0.40±(0.22)
	Σ 20	30.00%	7	1.17±(0.17)	1–2	0.35±(0.13)
*Anacridium aegyptium* (Linnaeus, 1764)^81^	♂ 7	28.57%	15	7.5±(3.5)	4–11	2.14±(1.58)
	♀ 1	0.00%	0	–	–	0
	Σ 8	25.00%	15	7.5±(3.5)	4–11	1.88±(1.39)
*Calliptamus barbarus* (Costa, 1836)^71^	♂ 153	62.75%	367	3.82±(0.34)	1–14	2.40±(0.26)
	♀ 111	74.77%	1161	13.99±(2.20)	1–103	10.46±(1.74)
	Σ 264	67.80%	1528	8.54±(1.10)	1–103	5.79±(0.78)
*Calliptamus coelesyriensis* Giglio-Tos, 1893	♂ 3	100.00%	17	5.67±(0.33)	5–6	5.67±(0.33)
	♀ 3	100.00%	25	8.33±(0.88)	7–10	8.33±(0.88)
	Σ 6	100.00%	42	7.00±(0.73)	5–10	7.00±(0.73)
*Calliptamus* spp.	14 immatures	71.43%	61	6.10±(1.52)	1–14	4.69±(1.37)
*Calliptamus tenuicercis* Tarbinsky, 1930	♂ 15	53.33%	15	1.88±(0.29)	1–3	1±(0.29)
	♀ 8	87.50%	89	12.71±(6.36)	1–45	11.13±(5.74)
	Σ 23	65.22%	104	6.93±(3.20)	1–45	4.52±(2.18)
*Charora persa rugosa* Bey-Bienko, 1951^82^	♂ 1	–	–	–	–	–
	♀ 0					
	*Σ* 1					
*Chorthippus (Glyptobothrus) brunneus* (Thunberg, 1815)^83^	♂ 38	13.16%	9	1.8±(0.49)	1–3	0.24±(0.12)
	♀ 18	11.11%	5	2.50±(0.50)	2–3	0.28±(0.19)
	Σ 56	12.50%	14	2±(0.38)	1–3	0.25±(0.10)
*Chorthippus* (*Chorthippus*) *dorsatus* (Zetterstedt, 1821)^75^	♂ 62	14.52%	20	2.22±(0.55)	1–6	0.32±(0.12)
	♀ 57	33.33%	76	4±(1.56)	1–30	1.33±(0.57)
	Σ 119	23.53%	96	3.43±(1.07)	1–30	0.81±(0.28)
*Chorthippus* spp.	♂ 24	33.33%	17	2.13±(0.29)	1–3	0.71±(0.23)
	♀ 29	62.07%	120	6.67±(1.31)	1–17	4.14±(1.01)
	Σ 53	49.06%	137	5.27±(1.00)	1–17	2.58±(0.61)
*Dociostaurus (Stauronotulus) hauensteini farsistanicus* Descamps, 1967^62^	♂ 10	70.00%	42	6.00±(1.89)	2–12	4.2±(1.58)
	♀ 22	68.18%	203	13.53±(2.55)	2–28	9.23±(2.20)
	Σ 32	68.75%	245	11.14±(1.97)	2–28	7.66±(1.63)
*Dociostaurus* (*Kazakia*) *tartarus* Stshelkanovtzev, 1921	♂ 29	24.14%	8	1.14±(0.14)	1–2	0.28±(0.09)
	♀ 30	23.33%	12	1.71±(0.29)	1–3	0.40±(0.15)
	Σ 59	23.73%	20	1.43±(0.17)	1–3	0.34±(0.90)
*Duroniella iranica *Bey-Bienko, 1948^84^	♂ 2	–	–	–	–	–
	♀ 4					
	Σ 6					
*Egnatioides farsistanicus* Uvarov, 1933^73^	♂ 10	30.00%	6	2.00±(0.58)	1–3	0.60±(0.34)
	♀ 13	61.54%	10	1.25±(0.16)	1–2	0.77±(0.20)
	Σ 23	47.83%	16	1.45±(0.21)	1–3	0.70±(0.18)
*Helioscirtus moseri* Saussure, 1884^65^	♂ 0	–	–	–	–	–
	♀ 3					
	Σ 3					
*Heteracris adspersa* (Redtenbacher, 1889)^85^	♂ 9	55.56%	6	1.20±(0.20)	1–2	0.67±(0.24)
	♀ 19	31.58%	8	1.33±(0.33)	1–3	0.42±(0.18)
	Σ 28	39.29%	14	1.27±(0.19)	1–3	0.50±(0.14)
*Heteracris littoralis* (Rambur, 1838)^86^	♂ 7	–	–	–	–	–
	♀ 3					
	*Σ* 10					
*Heteracris persa* (Uvarov, 1933)^73^	♂ 6	50.00%	4	1.33±(0.33)	1–2	0.67±(0.33)
	♀ 3	66.67%	9	4.5±(2.5)	2–7	3.00±(2.08)
	Σ 9	55.56%	13	2.60±(1.12)	1–7	1.44±(0.74)
*Heteracris pterosticha* (Fischer von Waldheim, 1833)^67^	♂ 17	11.76%	9	4.5±(3.5)	1–8	0.53±(0.47)
	♀ 11	9.09%	1	1	1	0.09±(0.09)
	Σ 28	10.71%	10	3.33±(2.33)	1–8	0.36±(0.29)
*Leptopternis gracilis* (Eversmann, 1848)^87^	♂ 1	–	–	–	–	–
	♀ 3					
	Σ 3					
*Mioscirtus wagneri* (Eversmann, 1859)^88^	♂ 42	19.05%	15	1.88±(0.40)	1–4	0.36±(0.14)
	♀ 37	45.95%	29	1.71±(0.19)	1–3	0.78±(0.17)
	Σ 79	31.65%	44	1.76±(0.18)	1–4	0.56±(0.11)
*Notostaurus anatolicus* (Krauss, 1896)	♂ 132	57.58%	320	4.21±(0.49)	1–27	2.42±(0.33)
	♀ 74	58.11%	345	8.02±(1.98)	1–76	4.66±(1.23)
	Σ 206	57.77%	665	5.59±(0.80)	1–76	3.23±(0.49)
*Notostaurus* spp.	11 immatures	100.00%	26	2.36±(0.34)	1–4	2.36±(0.34)
*Ochrilidia gracilis* (Krauss, 1902)^89^	♂6	–	–	–	–	–
	♀ 7					
	Σ 13					
*Oedipoda kurda* Descamp, 1967^62^	♂ 27	55.56%	42	2.80±(0.51)	1–8	1.56±(0.39)
	♀ 14	85.71%	39	3.25±(0.71)	1–10	2.79±(0.68)
	Σ 41	65.85%	81	3.00±(0.42)	1–10	1.98±(0.35)
*Oedipoda miniata* (Pallas, 1771)^63^	♂ 240	51.67%	410	3.31(± 0.3)	1–28	1.71±(1.88)
	♀ 270	61.85%	676	4.05±(0.39)	1–48	2.5±(0.27)
	Σ 510	57.06%	1086	3.74±(0.25)	1–48	2.13±(0.17)
*Oedipoda* spp.	3 immatures	100.00%	9	3.33±(1.20)	1–5	3.33±(1.20)
*Pseudoceles dirshi* Popov, 1951^90^	♂ 7	42.86%	11	3.67±(1.33)	1–5	1.57±(0.90)
	♀ 7	85.71%	17	2.83±(0.60)	1–5	2.43±(0.65)
	Σ 14	64.29%	28	3.11±(0.56)	1–5	2.00±(0.54)
*Pyrgodera armata* (Fischer von Waldheim, 1820)	♂ 7	71.43%	24	4.80±(2.58)	1–15	3.43±(1.99)
	♀ 7	71.43%	26	5.20±(2.37)	1–14	3.71±(1.90)
	Σ 14	71.43%	50	5.00±(1.65)	1–15	3.57±(1.32)
*Ramburiella* (*Palaeocesa*) *bolivari* (Kuthy, 1907)	♂ 2	50.00%	1	1	1	0.50±(0.50)
	♀ 1	100.00%	4	4	4	4
	Σ 3	66.67%	5	2.5±(1.50)	4	1.67±(1.20)
*Ramburiella* (*Palaeocesa*)	♂ 1	100.00%	2	2	2	2
	♀ 0	0.00%	0	0	–	0
*turcomana* (Fischer von Waldheim, 1833)^67^	Σ 1	100.00%	2	2	2	2.00
*Schistocerca gregaria* (Forskål, 1775)	♂ 0	0.00%	0	–	–	0
	♀ 1	100.00%	1	1	1	1
	Σ 1	100.00%	1	1	1	1.00
*Sphingoderus angustus* Descamps, 1967^62^	♂ 7	14.29%	1	1	1	0.14±(0.14)
	♀ 9	33.33%	7	1	1–5	0.78±(0.55)
	Σ 16	25.00%	8	2±(1.00)	1–5	0.50±(0.32)
*Sphingoderus carinatus* (Saussure, 1888)^64^	♂ 37	24.32%	21	2.33±(0.65)	1–7	0.57±(0.22)
	♀ 23	30.43%	15	2.14±(1.33)	1–5	0.65±(0.28)
	Σ 60	26.67%	36	2.25±(0.44)	1–7	0.60±(0.17)
*Sphingonotus* (*Sphingonotus*) *coerulipes* Uvarov, 1922	♂ 23	56.52%	20	1.54±(0.27)	1–4	0.87±(0.22)
	♀ 13	69.23%	26	2.89±(0.51)	1–5	2.00±(0.52)
	Σ 36	61.11%	46	2.09±(0.29)	1–5	1.28±(0.25)
*Sphingonotus* (*Sphingonotus*) *nebulosus persa* Saussure, 1884^65^	♂ 9	77.78%	17	2.43±(0.57)	1–5	1.89±(0.56)
	♀ 3	100.00%	10	3.33±(1.20)	1–5	3.33±(1.20)
	Σ 12	83.33%	27	2.7±(0.52)	1–5	2.25±(0.52)
*Sphingonotus* (*Sphingonotus*) *pilosus* Saussure, 1884	♂ 43	39.53%	38	2.24±(0.42)	1–7	0.88±(0.24)
	♀ 28	42.86%	27	2.25±(0.65)	1–7	0.96±(0.35)
	Σ 71	40.85%	65	2.24±(0.36)	1–7	0.92±(0.20)
*Sphingonotus (Sphingonotus) rubescens* (Walker, 1870)^72^	♂ 28	28.57%	19	2.38±(0.96)	1–9	0.68±(0.33)
	♀ 34	44.12%	37	2.47±(0.61)	1–8	1.09±(0.34)
	Σ 62	37.10%	56	2.43±(0.51)	1–9	0.90±(0.24)
*Sphingonotus* (*Sphingonotus*) *satrapes* Saussure, 1884^65^	♂ 9	11.11%	1	1	1	0.11±(0.11)
	♀ 4	25.00%	1	1	1	0.25±(0.25)
	Σ 13	15.38%	2	1	1	0.15±(0.10)
*Sphingonotus* (*Sphingonotus*) *savignyi* Saussure, 1884^65^	♂ 2	0.00%	0	–	–	0
	♀ 7	14.29%	2	2	2	0.29±(0.29)
	Σ 9	11.11%	2	2	2	0.22±(0.22)
*Sphingonotus* sp.	3 immatures	100.00%	8	2.67±(1.20)	1–5	2.67±(1.20)
*Sphingonotus* (*Sphingonotus*) *theodori* Uvarov, 1923	♂ 56	41.07%	77	3.35±(0.79)	1–16	1.38±(0.39)
	♀ 63	39.68%	104	4.16±(0.68)	1–13	1.65±(0.37)
	Σ 119	40.34%	181	3.77±(0.52)	1–16	1.52±(0.27)
*Sphodromerus luteipes* Uvarov, 1933^73^	♂ 30	53.33%	100	6.25±(1.82)	1–32	3.33±(1.12)
	♀ 12	75.00%	27	3.00±(0.53)	1–5	2.55±(0.55)
	Σ 42	59.52%	127	5.08±(1.21)	1–32	3.02±(0.81)
*Stenohippus xanthus* (Karny, 1907)	♂ 18	22.22%	5	1.250. (0.25)	1–2	0.28±(0.13)
	♀ 13	69.23%	20	2.22±(0.55)	1–5	1.54±(0.47)
	Σ 31	41.94%	25	1.92±(0.40)	1–5	0.81±(0.24)
*Truxalis eximia* Eichwald, 1830	♂ 1	100.00%	3	3	3	3
	♀ 0	0.00%	0	–	–	0
	Σ 1	100.00%	3	3	3	3.00
*Truxalis mesopotamica* Dirsh, 1950^76^	♂ 12	58.33%	42	6.00±(4.02)	1–30	3.5±(2.44)
	♀ 6	50.00%	33	11.00±(6.00)	5–23	5.50±(3.64)
	Σ 18	55.56%	75	7.50±(3.25)	1–30	4.17±()1.98
*Truxalis robusta* (Uvarov, 1916)	♂ 3	66.67%	46	23	23	15.33±(7.67)
	♀ 0	0.00%	0	–	–	0
	Σ 3	66.67%	46	23	23	15.33±(7.67)
Total	♂1266	43.36%	1882	3.43±(0.16)	1–32	1.49±(0.08)
	♀1072	52.24%	3353	5.99±(0.44)	1–103	3.13±(0.25)
	32 immatures	87.50%	109	3.89±(0.65)	1–14	3.41±(0.61)
	Σ 2370	47.97%	5344	4.70±(0.23)	1–103	2.25±(0.12)

Attachment sites were categorized into 15 regions: 1- hindwing base, 2- hindwing end, 3- tegmina, 4- wingpad, 5- pronotum, 6-mesothorax, 7-metathorax, 8-sternum, 9- tympanum,10- abdomen, 11- coxa, 12- femur, 13- tibia, 14- tarsus and 15- head.

## Results

### Differences between years

Sampling was conducted over two years (2020 and 2021), reported 1,620 grasshoppers and 3,693 mites in the first year, and 750 grasshoppers and 1651 mites in the second year. However, the difference in mean intensity between years was not statistically significant (Mann–Whitney U test, *p* = 0.123).

### Frequency, prevalence and intensity

A total of 5344 mites were collected from 2370 grasshopper individuals. The grasshoppers belonged to 48 species; among these, *Chorthippus* (*Chorthippus*) *dorsatus* (Zetterstedt, 1821)^75^ was recorded for the first time for the grasshopper fauna of Iran. For mites, 13 species were identified.

**Fig. 3:**
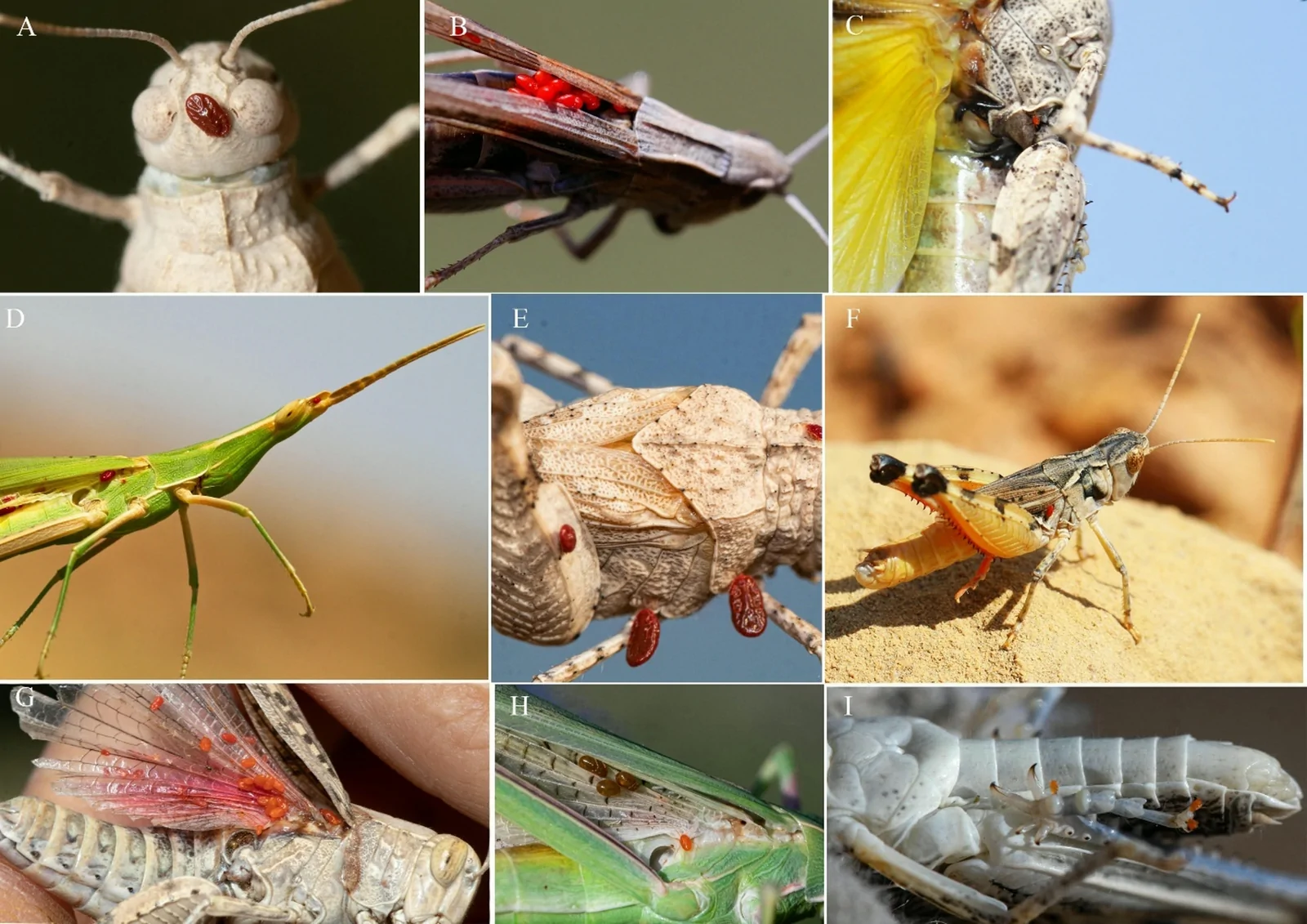
Parasitic mites attached to their grasshopper hosts: (**A**)* Leptus (Leptus) darvishi on Oedipoda miniata*; (**B**) *Eutrombidium cf. trigonum on Chorthippus (Chorthippus) dorsatus*; (**C**)* Charletonia ahwazensis on Mioscirtus wagneri,* (**D**) *Charletonia baluchestanica on Acrotylus oxycephala,* (**E**) *Abalakeus lorestanicus on Oedipoda miniata;* (**F**) *Leptus (Leptus) darvishi on Dociostaurus hauensteini farsistanicus;* (**G**) *Eutrombidium sorbasiensis on Calliptamus barbarus;* (**H**) *Charletonia krendowskyi on Truxalis mesopotamica; I. Tafreshichyzeria hessabyi on Notostaurus* spp., Field photographs taken by the first author (**B**,**H**,**I**) and the fourth author (**A**,**C**,**D**,**E**,**F**,**G**).

Grasshopper samples included 1266 males, 1072 females and 32 immatures. Males were more significantly more frequently collected than females (Table 4, Chi-square test, *p* < 0.001). The overall infestation rate was 47.97%, with a significantly higher prevalence in females (52.24%) than in males (43.36%) (*p* < 0.001). The highest infestation intensity was observed on *Calliptamus barbarus* (Costa, 1836)^71^, which harbored 103 individuals of *Eutrombidium* cf. *trigonum.* Larger hosts, such as those in the subfamily Calliptaminae, tended to carry heavier parasite loads, and high mite intensity was observed particularly on the wings (Fig. 3G).

The highest prevalence (excluding hosts represented by fewer than three individuals) was found in *Sphingonotus* (*Sphingonotus*) *nebulosus persa* Saussure, 1884, with 83.33%. The highest mean intensity (11.14) was recorded for *Dociostaurus* (*Stauronotulus*) *hauensteini farsistanicus* Descamps, 1967^62^ (Fig. 3F). No mites were collected from *Ochrilidia gracilis* (Krauss, 1902)^89^, *Charora persa rugosa* Bey-Bienko, 1951^82^, *Duroniella iranica* Bey-Bienko, 1948^84^, *Helioscirtus moseri* Saussure, 1884, *Leptopternis gracilis* (Eversmann, 1848)^87^, and *Heteracris littoralis* (Rambur, 1838)^86^ (Table 4).

**Fig. 4:**
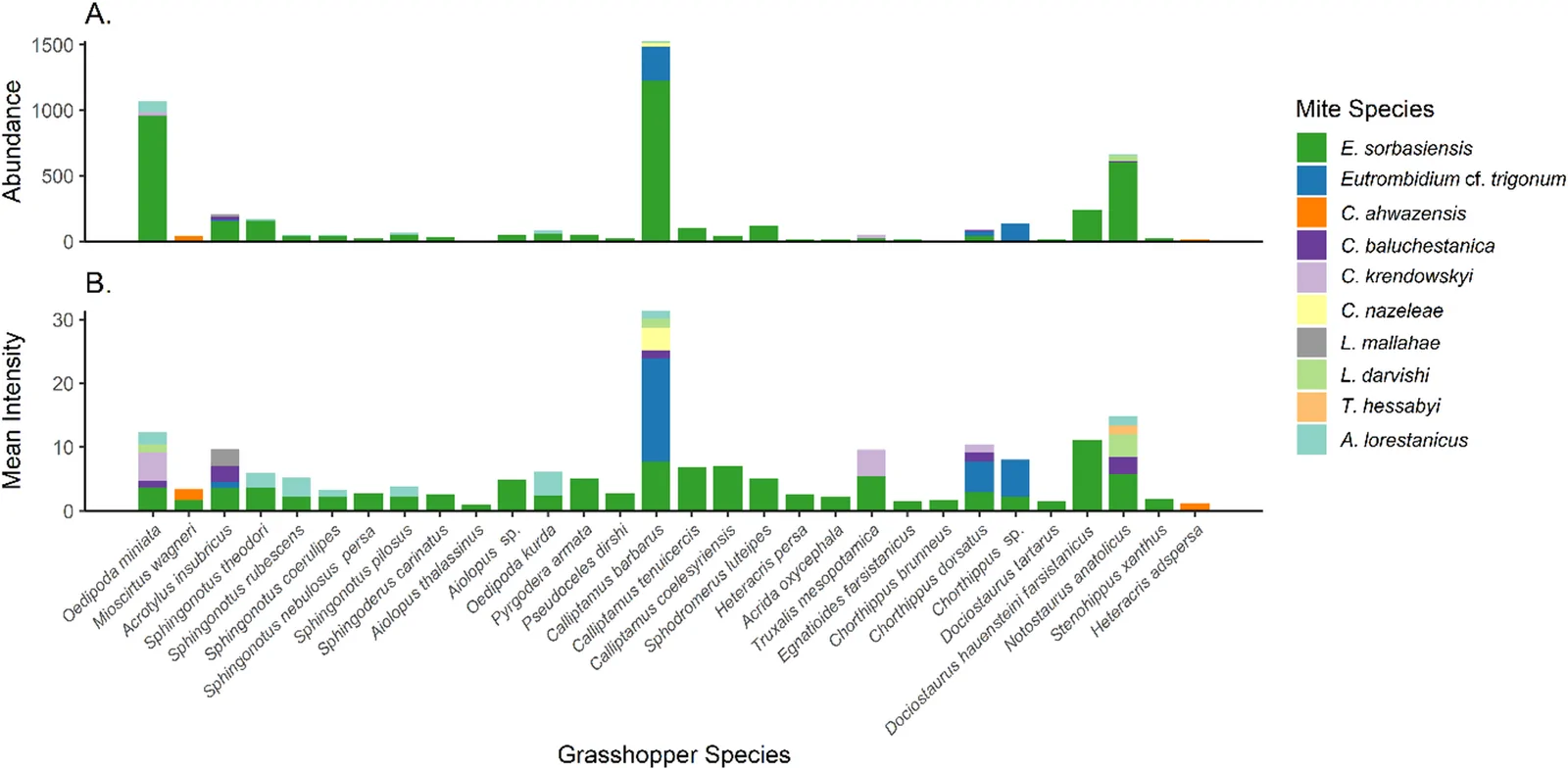
Mite species abundance and mean intensity in their grasshopper hosts.

The most abundant mite species was *Eutrombidium sorbasiensis* Mayoral & Barranco, 2004^70^ (*N* = 4282), with the highest number (*N* = 1222) collected from *Calliptamus barbarus* (Fig. 4A).

The maximum mean intensity was observed for *Eutrombidium* cf. *trigonum* (Hermann, 1804)^74^ from *Calliptamus barbarus*, (16.25) and for *Eutrombidium sorbasiensis* from *Dociostaurus* (*Stauronotulus*) *hauensteini farsistanicus* (11.05). After excluding mites with fewer than three host individuals, the following mean intensities were noted (Fig. 4B): *Charletonia ahwazensis* on *Mioscirtus wagneri* (Eversmann, 1859)^88^: 1.79; *Charletonia baluchestanica* on *Notostaurus anatolicus* (Krauss, 1897)^91^: 2.67; *Charletonia krendowskyi* on *Oedipoda miniata* (Pallas, 1771)^63^: 7.5; *Charletonia nazeleae* on *Calliptamus barbarus*: 3.5; *Leptus* (*Leptus*) *mallahae* on *Acrotylus insubricus* (Scopoli, 1786)^69^: 2.75; *Leptus* (*Leptus*) *darvishi* on *Notostaurus anatolicus*: 3.58; *Tafreshichyzeria hessabyi* on *Notostaurus* sp.: 1.57 and *Abalakeus lorestanicus* on *Oedipoda kurda* Descamps, 1967^62^: 3.86.

### Multi-parasitism

**Fig. 5:**
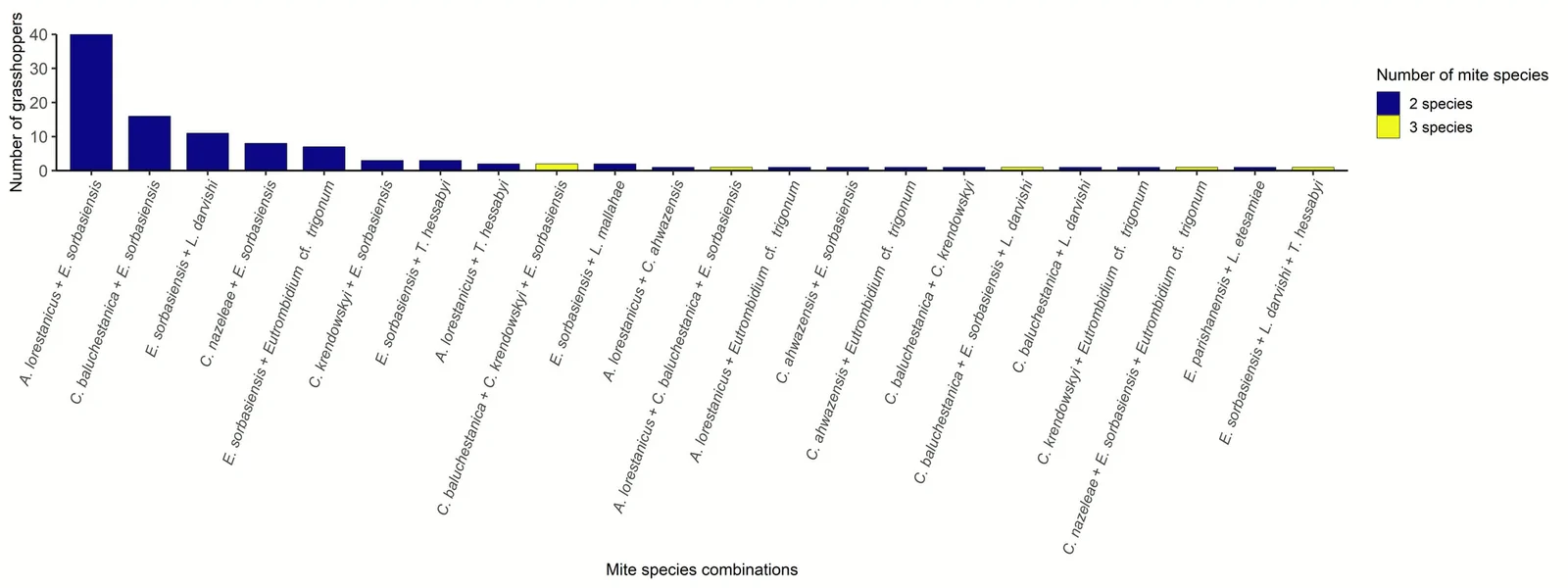
Combinations of mite species in multiparasitized grasshoppers.

Out of 1,137 infested hosts, 101 (8.95%) were parasitized by two mite species, and six (0.05%) by three (Fig. 5).

Most multi-parasitized hosts were *Oedipoda miniata* (*N* = 29), *Calliptamus barbarus* (*N* = 19), and *Acrotylus insubricus* (*N* = 13). Combinations of mite species in multi-parasitized grasshoppers are also shown in Fig. 5.

### Host associations and the bipartite network

**Fig. 6:**
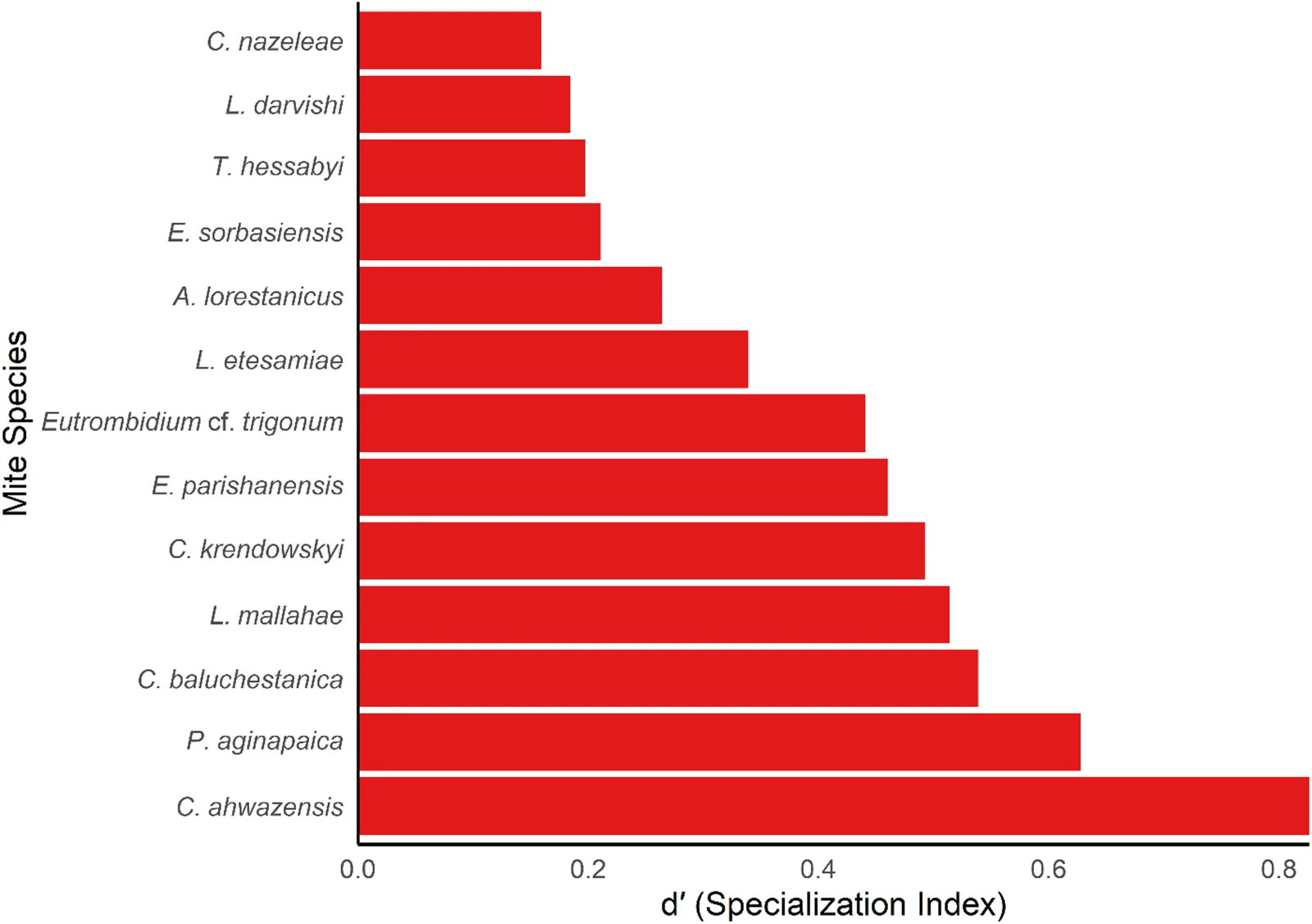
Mite species d′ (degree of host specificity).

The bipartite network (Fig. 2) revealed interactions between 42 grasshopper species and 13 mite species. Link width in the network indicates interaction strength based on the abundance of mite individuals on each host species. The network-level specialization index (H_2_′) was 0.376, indicating a moderate level of specialization, while connectivity was relatively low at 21%. Species-level specialization (d′) ranged from 0.16 to 0.83, with *Charletonia ahwazensis* showing the highest host specificity (d′ = 0.83). Overall, the plot indicates a generalist pattern of parasitism (Fig. 6).

### Elevation and habitat effects on mite abundance

Dunn’s post hoc test (Table 2) revealed significant altitudinal preferences for several mite species: *Eutrombidium sorbasiensis* had a wide altitudinal range but was significantly more abundant in certain hosts at specific altitudes: *Oedipoda miniata*, *Sphingonotus* (*Sphingonotus*) *theodori* Uvarov, 1923 in Buxton and Uvarov^92^, *Sphingonotus* (*Sphingonotus*) *rubescens* (Walker, 1870)^72^ and *Sphingoderus carinatus* (Saussure, 1888)^64^ at 800–1200 m; *Acrotylus insubricus* and *Calliptamus barbarus* at 1600–2000 m; *Sphodromerus luteipes* Uvarov, 1933^73^ and *Notostaurus anatolicus* above 2000 m.

*Eutrombidium* cf. *trigonum* was most abundant in the Arjan Lagoon (> 2000 m); significant differences were observed across five hosts (*p* < 0.04). The larvae were identified as *Eutrombidium* cf. *trigonum* based on their close morphological resemblance to *Eutrombidium trigonum* (Hermann, 1804)^74^. Our specimens, however, have two deviations from *Eutrombidium trigonum*: (1) presence of reticulation on the scutum and scutellum, which has not been previously documented for this species, and (2) an SA/SP ratio < 1.5. Measurements are provided in (Supplementary Table 2). *Abalakeus lorestanicus* showed a preference for 1200–1600 m in *Sphingonotus* (*Sphingonotus*) *pilosus* Saussure, 1884 and *Oedipoda kurda*, and 1600–2000 m in *Oedipoda miniata* and *Sphingoderus carinatus*. *Charletonia krendowskyi* preferred elevations > 2000 m, especially on *Acrotylus insubricus* (*adj. p* = 0.03–0.08), whereas *Charletonia ahwazensis* was more abundant at 1200–1600 m on *Heteracris pterosticha* (Fischer von Waldheim, 1833)^67^ (*adj. p* < 0.000001).

### Habitat preferences

The post hoc Dunn’s tests (Table 3) provided several significant results: *Eutrombidium sorbasiensis* was significantly more abundant in forest-steppe habitats across seven host species (*p* ≤ 0.04). *Eutrombidium* cf. *trigonum* favored wetland and riparian zones when parasitizing *Sphingoderus carinatus*. *Charletonia krendowskyi* showed a significant preference for mountainous habitats when on *Chorthippus* (*Glyptobothrus*) *brunneus* (Thunberg, 1815)^83^ and *Chorthippus* (*Chorthippus*) *dorsatus* (Zetterstedt, 1821)^75^. *Charletonia baluchestanica* preferred forest steppe and foothills in *Chorthippus* (*Chorthippus*) *dorsatus*. *Leptus* (*Leptus*) *mallahae* favored *Acrotylus insubricus* in plains. *Tafreshichyzeria hessabyi* was preferably found on *Notostaurus anatolicus* in forest-steppes compared to wetland and riparian habitats (*Z* = 2.79, *adj. p* = 0.032).

## Discussion

Despite the increasing number of studies investigating host-parasite relationships, our knowledge of the host ranges of Parasitengona mites remains limited. This is partly related to the lack of ecological studies on parasite species and the inadequate identification of many hosts. In this study we attempt to contribute to this topic: we collected 48 species of grasshoppers and 13 species of mites from 22 locations across five transects in the Zagros mountains of Iran; this represents nearly one quarter of the Acrididae species reported from the country to date^26^. The overall prevalence of mite infestation across all collected specimens was 47.97%, a notably high rate compared to previous studies^93, 9, 94, 34^. In the following, we discuss the results of our analyses in detail.

### Mite diversity and host associations

Severin,^95^ observed that over 90% of mites parasitizing grasshoppers belonged to the genus *Eutrombidium*, which is consistent with our findings. Members of this genus are distributed across all continents except Antarctica. According to Anderson^93^, *Eutrombidium* species infest their hosts more intensively compared to other genera, such as *Leptus*, which can partly explain the high parasite burden of *Eutrombidium* species compared to other genera. *Leptus* Latreille, 1796 (Acari, Prostigmata, Erythraeidae) species infest their hosts in lower numbers compared to *Eutrombidium* Verdun, 1909^93^. For example, the highest intensity of *Leptus* (*Leptus*) *darvishi* Saboori, Hakimitabar & Khademi, 2018 (Fig. 3A) was 10 individuals on *Notostaurus anatolicus* (Krauss, 1897)^91^. In contrast, the genus *Charletonia* showed intermediate intensity, with the highest record being 23 individuals of *Charletonia baluchestanica* Tashakor & Hakimitabar, 2015 from *Acrida oxycephala* (Pallas, 1771)^63^ (Fig. 3D). Members of Erythraeidae, especially of the genus *Leptus*, generally have a wide host range and infest many insect orders^96, 97, 98^.

About 80% of the collected mites were *Eutrombidium sorbasiensis*, a species known for its adaptability to various hosts and habitats and a wide distribution in many regions of Europe to Iran^49^. Up to now, Acrididae are the only hosts of this species^99, 100^. A total of 4,282 *Eutrombidium sorbasiensis* individuals were collected with infestation intensities ranging from 1 to 96. This species was absent only from eight host species, most of which were rare in the sampling area and subsequently had fewer chances for infestation. The two most heavily infested grasshopper species were *Calliptamus barbarus* (mean intensity: 7.69) and *Oedipoda miniata* (mean intensity: 3.65), which were also the most abundant hosts. This pattern is consistent with the general expectation that parasites tend to be more successful on common host species^101^. Other commonly collected species included *Eutrombidium* cf. *trigonum* (481 individuals) and *Abalakeus lorestanicus* (180 individuals). *Eutrombidium* cf. *trigonum* was found in only five habitats and on a small range of hosts. The overall morphology of the examined specimens is consistent with *Eutrombidium trigonum*, supporting their tentative identification as *Eutrombidium* cf. *trigonum*. Historically, the SA/SP ratio was used to distinguish *E. locustarum* from *E. trigonum*; however, the validity of this character is contested following their synonymization^49^, suggesting this character alone may not be taxonomically reliable and hence the taxonomic status still remains unresolved^102, 103, 17, 104^.

*Abalakeus lorestanicus* had a broader distribution and host range. This species has been documented both in Iran and Spain, always from Orthopteran hosts^70, 105, 55^. *Charletonia nazeleae* Karimi Iravanlou, Kamali & Talebi, 2002, *Leptus* (*Leptus*) *darvishi* Saboori, Hakimitabar & Khademi, 2018, *Charletonia ahwazensis* Haitlinger & Saboori, 2007^66^, *Tafreshichyzeria hessabyi* Saboori and Lazarboni, 2007^56^, *Charletonia baluchestanica* Tashakor & Hakimitabar, 2015 and *Charletonia krendowskyi* (Feider, 1954)^68^, depending on their presence in the habitat, infested the range of grasshoppers (Fig. 3).

### Mite abundance and habitat associations

The mean abundance of the six mite species collected across different altitudes varied significantly (Table 2). This variation may be attributed to the altitude-specific distributions of both the mites and their grasshopper hosts. For example, *Charletonia ahwazensis* was primarily collected at lower altitudes (1200–1600 m), particularly from the Kaftarak station, which is adjacent to Maharloo Lake, a hypersaline wetland. Although no previous information exists about the habitat preferences of this species, our findings suggest that it may be tolerant of hypersaline soils. Notably, *Charletonia ahwazensis* was predominantly collected from two grasshopper species – *Mioscirtus wagneri* (34 specimens) and *Heteracris adspersa* (11 specimens) – both of which are known to inhabit saline environments.

**Fig. 7:**
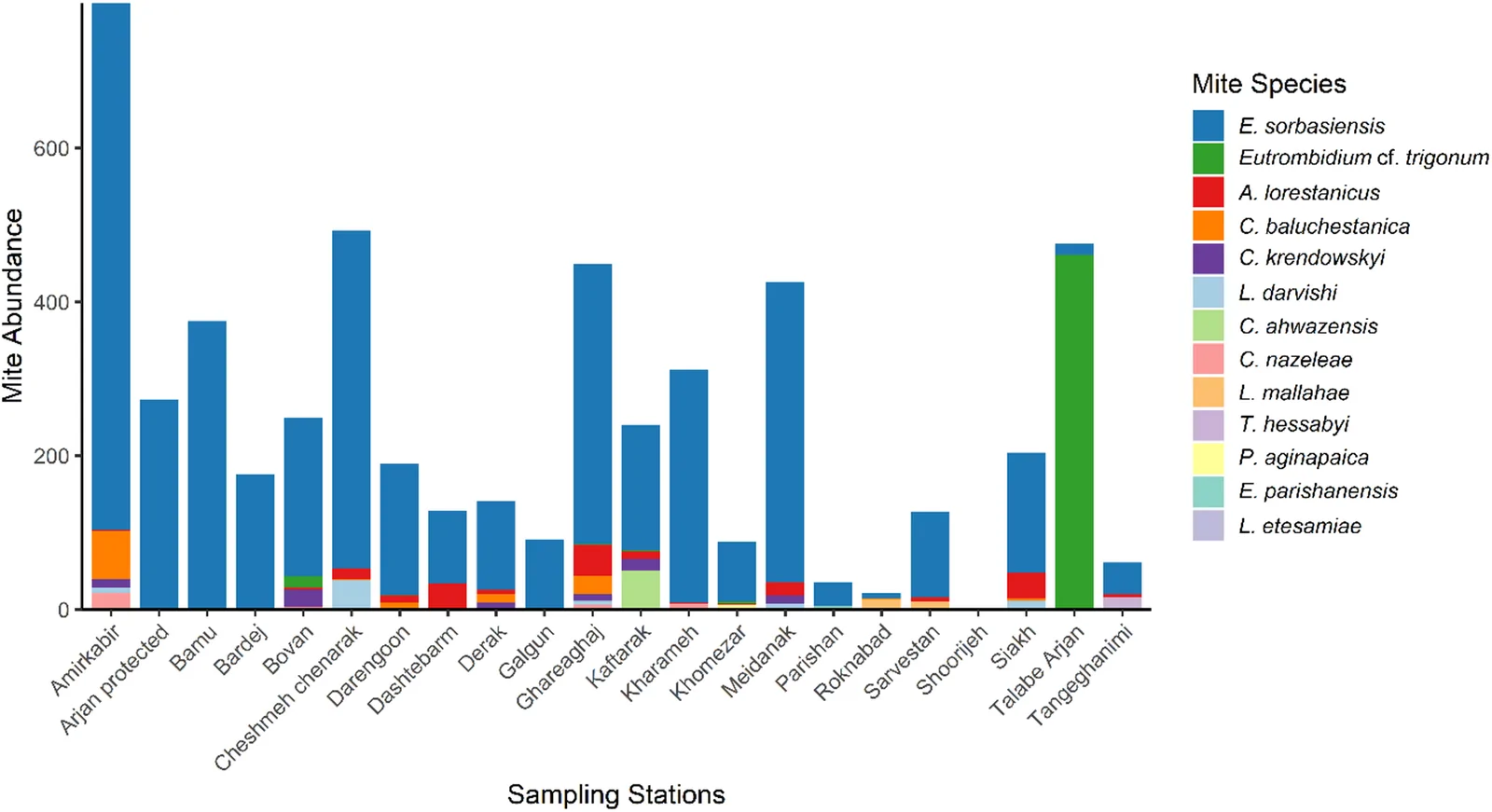
Mite species abundance on each station.

*Eutrombidium sorbasiensis* was significantly more abundant in forest-steppe habitats (Table 3), likely due to the higher humidity levels characteristic of these areas. This species was particularly prevalent at the Amirkabir station (686 specimens), a foothill region with diverse vegetation cover. In contrast, *Eutrombidium* cf. *trigonum*, primarily collected from *Sphingoderus carinatus*, exhibited a strong preference for riparian and wetland habitats, especially Arjan station, a lagoon environment where this species was found in large numbers (Fig. 7).

According to Sevsay and Karakurt^103^; this species inhabits grasslands near lakes and other aquatic habitats, which is consistent with our findings. Interestingly, while *Eutrombidium cf. trigonum* was abundant in the moist soil of Arjan Lagoon, *Eutrombidium sorbasiensis* was rare in the same area. Such contrast may reflect interspecific competition or differences in tolerance to local microhabitat conditions. Similar patterns have been noted in previous studies^106, 8^, which suggest that habitat can play an important role in structuring the distribution of some Parasitengona taxa. Nevertheless, the relative importance of habitat conditions and host associations likely varies among species and taxonomic groups, indicating that these relationships should be interpreted in a taxon-specific ecological context.

At arid stations such as Shoorijeh and Roknabad, we recorded only very few *Eutrombidium sorbasiensis* specimens, possibly reflecting this species’ sensitivity to drought. Trombidioid mites, unlike erythraeids, are relatively vulnerable to desiccation; their eggs dry up and die when soil humidity drops, indicating poor drought resistance^107^. In contrast, erythraeids possess several adaptations, such as an additional sheath covering their eggshell and the absence of some body openings including Claparède’s organs and an anal opening allowing them to withstand dry conditions^108, 107^.

However, despite the interesting results, several limitations in our analyses should be noted. Although our sampling design aimed to cover diverse altitudinal ranges and habitat types separately, these two factors can naturally overlap in complex mountain landscapes like the Zagros Mountains. While similar habitats were observed across different elevation bands, minimizing the risk of confounding effects, the potential for localized micro-environmental effects remains. Furthermore, as sampling stations were not explicitly modeled as random effects, observations within the same site may not be fully independent.

### Gender bias

Numerous studies have investigated sex-biased parasitism in Parasitengona hosts with varying results. Some studies report higher infestation in males^93, 109, 101, 8^), while others find females more heavily infested^10, 110, 111^. In our study, females had a higher mean infestation intensity than males, possibly due to their larger size and higher nutritional value providing more hemolymph and fat reserves for egg production^93^. Furthermore, it is plausible that female grasshoppers, due to their role in oviposition, provide ecological advantages to mites. Engorged mites may detach during egg-laying and remain near the egg pods, feeding on them during subsequent developmental stages – a behavior reported in previous studies^93, 95, 112^.

In the sexually dimorphic Calliptaminae subfamily (*Calliptamus barbarus*, *Calliptamus tenuicercis*, *Sphodromerus luteipes*), females were significantly more parasitized than males. For example, *Calliptamus barbarus* had a mean female infestation rate of 13.99 compared to 3.82 in males. As a widespread species across Europe and Asia^47^, the large-bodied female *Calliptamus barbarus* likely provides more resources for mites.

### Attachment sites

Previous studies suggested that nymphs, wingless or short-winged grasshoppers are less frequently parasitized^9, 113, 95^. This may be because winged, mature grasshoppers play a more crucial role in expanding the geographic distribution of Parasitengona by accessing distant habitats^6^. The wings can also act as a shelter and offer protection to mites from certain environmental factors.

**Fig. 8:**
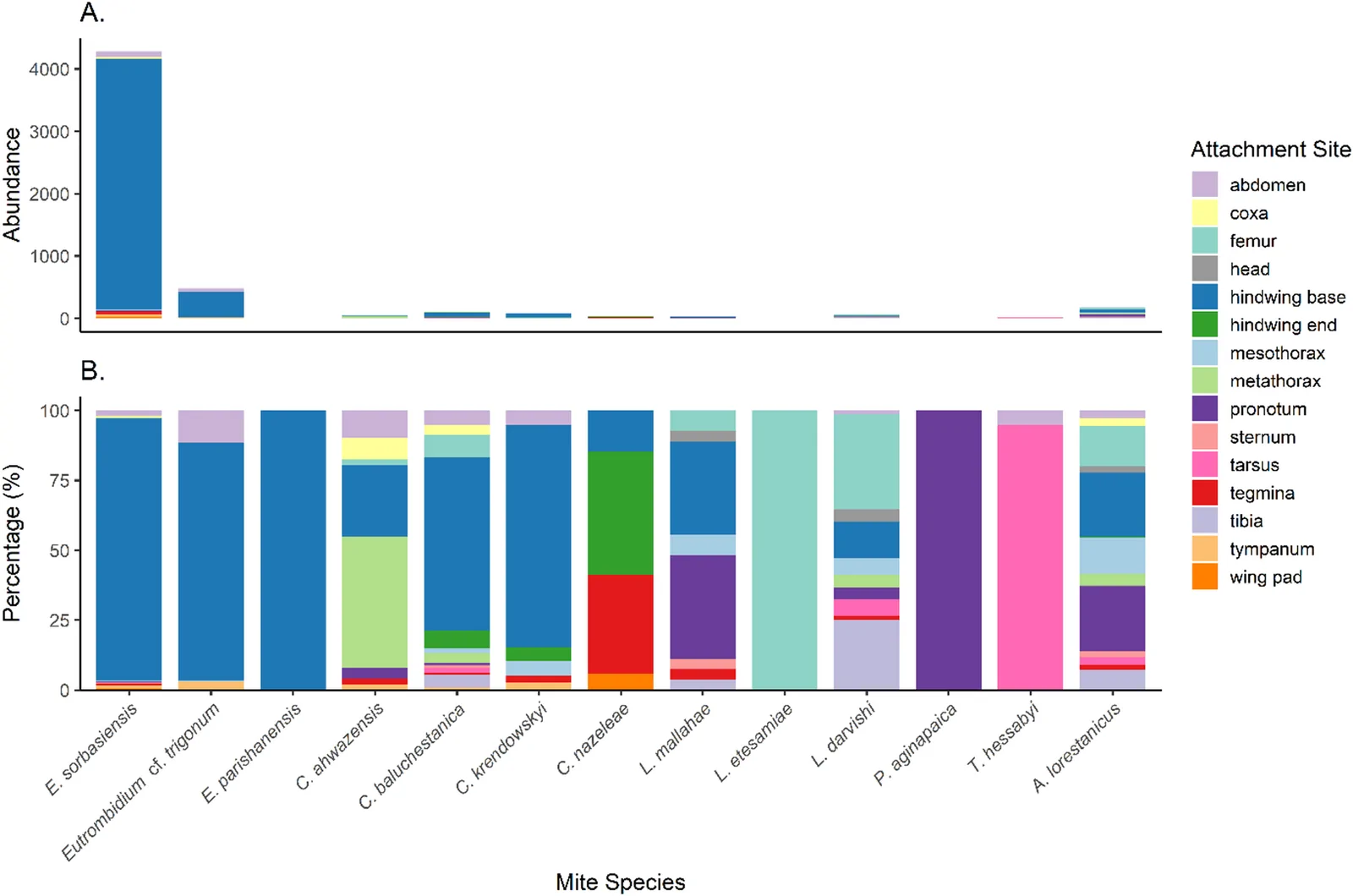
Attachment sites preferences of mite species: (**A**) Mites abundance on different appendages; (**B**) mites percentage on different appendages.

Generally, Parasitengona larvae were attached to various structures on grasshopper hosts, but some species showed clear site preferences. *Eutrombidium sorbasiensis*, for example, consistently preferred the hindwing base across all adult hosts (Fig. 8A-B, Supplementary Table 1).

Nymphs, lacking developed hindwings, showed attachment to alternative sites such as wing pads, tympana, and femora. This is consistent with Severin^95^, who reported *Eutrombidium* attached to wing pads in nymphs and wings in adults. Anderson,^93^ similarly noted a preference for hindwings and wing pads by *Eutrombidium*. The hindwing base may offer several advantages to mites: protection from host grooming and environmental disturbance, as well as access to higher hemolymph flow, especially near the wing base^114^, thereby enhancing nutrient intake. Accordingly, also Welbourn^115^ reported that *Eutrombidium robauxi* preferentially attached to hindwings, when hosts were adults.

Nonetheless, alternative attachment sites were recorded, such as the prothorax^116^, leg insertions and softer intersegmental regions^112^. Our study is in line with these previous results; 1.9% of *Eutrombidium sorbasiensis* was found on the abdomen and 0.08% on leg joints. *Eutrombidium* cf. *trigonum* exhibited the same hindwing base preference; however, Husband and Wohltmann,^102^ mentioned *E. trigonum* mostly attaches to the soft cuticle of the host’s thorax and rarely attaches to the wings, and *E. locustarum* shows the opposite pattern, with a marked preference for the hindwings of its hosts.

Among *Charletonia* species, *Charletonia krendowskyi* showed a significant preference for hindwing attachment in hosts such as *Oedipoda miniata*, *Truxalis mesopotamica* Dirsh, 1950^76^, *Anacridium aegyptium* (Linnaeus, 1764)^81^ and *Chorthippus* (*Chorthippus*) *dorsatus* (Zetterstedt, 1821)^75^. Similarly, *Charletonia baluchestanica* preferred the hindwing in *Oedipoda miniata*, *T. mesopotamica*, and *Acrotylus insubricus* (Scopoli, 1786)^69^. However, *Charletonia ahwazensis* displayed a different pattern, attaching predominantly to the metathorax in *Mioscirtus wagneri* and *Heteracris adspersa* (Redtenbacher, 1889)^85^. This variability within *Charletonia* aligns with some earlier reports; others yielded different results: Wohltmann et al.^112^ suggested non-selective attachment in *Charletonia cardinalis*, while Key^117^ and Key and Southcott^118^ found attachment primarily to the prothorax. Kekeunou et al.^94^ noted thoracic attachment predominance, possibly due to softer cuticle or mechanical shelter from wings. Similarly, Elverici et al.^119^ found all *Charletonia krendowskyi* mites on the hindwings of *Platycleis intermedia* (Serville, 1838). In *Tafreshichyzeria hessabyi*, a native species, 18 of 19 individuals were found attached to the tarsi, a preference not previously reported. While data are limited, this may reflect a true site preference.

*Leptus* (*Leptus*) *darvishi* demonstrated frequent attachment to the tibia in *Calliptamus barbarus* and *Notostaurus anatolicus*. Anderson^93^ noted that *Leptus* larvae commonly attach to the head, thorax, and legs. Prior studies have shown preferences for femur and tibia^120, 121, 122, 123^, which is supported by our findings. Some studies^124, 98, 125, 3, 112^, have reported that *Leptus* larvae do not show a clear preference for specific attachment sites and typically parasitize heavily sclerotized areas of the host’s body. They are not seen in the membranous intersegmental areas. However, some studies also indicate the tendency of members of this genus to attach to soft cuticle^109, 126, 127^. Interspecific variation likely plays a central role, influenced by host species’ morphology and grooming behavior.

When parasitizing *Oedipoda miniata*, *Abalakeus lorestanicus* attachment to the hindwing base was significantly more frequent than at other regions. Femora were also more frequently targeted than areas like the tympanum or abdomen. This species, however, showed host-dependent variation. For example, in *S.* (*S.*) *pilosus*, which has dense body hair that may hinder grooming, it was found attached to the femur, while in *S.* (*S.*) *coerulipes* and *S.* (*S.*) *theodori*, mesothorax and pronotum were preferred, respectively. In *Notostaurus anatolicus*, the attachment preference was the hindwing base, and in *Notostaurus* spp. (immature individuals) it was preferably found at the tarsus. This supports the hypothesis that grooming efficiency and host morphology also influence attachment sites. As noted by Kerry and Baker^128^ and Zajkowska and Mąkol^8^, grooming may dislodge larvae from accessible regions, leaving mites clustered on less reachable body parts.

Importantly, these patterns of attachment‑site variation provide the ecological context for understanding how multiple mite species may coexist on the same host individual. When multiparasitism occurs, differentiation in microhabitat use on the host body can act as a form of spatial niche partitioning, thereby mitigating potential interspecific competition. Thus, attachment‑site variation should be interpreted not only in the light of host morphology and grooming behavior, but also as a potential mechanism facilitating coexistence under multi-parasitic conditions.

### Patterns of multi-parasitism

Beyond sexual dimorphism, factors such as superparasitism – where parasites preferentially infest already parasitized hosts – may explain heavier parasite loads in some individuals^111, 129^. This may be due to reduced host defenses or easier accessibility, and it can also increase the chances of establishing new mite populations by ensuring that both sexes of mites are present. This may also apply across species.

While multi-parasitism is considered rare in terrestrial Parasitengona^6, 130^, it has been more frequently documented in aquatic mites^131, 132^. Based on Wohltmann^130^ and Felska et al.,^6^, Parasitengona with arthropod hosts choose a different range of hosts to reduce host competition; hence, co-parasitism of more than one species of mite on a single host is scarce and usually not found with closely related species of Parasitengona with arthropod host; however, it has been recorded from chiggers on mammal hosts^133,134^. According to our findings, it seems that mites in case of multi-parasitism chose different microhabitats at the host’s body to attach. In over 73% of multi-parasitized hosts, mites occupied different body regions. Specifically, in hosts with both *Eutrombidium* and *Charletonia*, the mites often selected different attachment sites, e.g. the hindwing base versus the hindwing tip, suggesting niche partitioning to minimize interspecific competition. In single infestations, *Charletonia* usually favored the hindwing base, but shifted position in the presence of *Eutrombidium*. This pattern indicates spatial partitioning of the host body regions by mite species and is consistent with the concept of niche partitioning. When two species from the same genus co-occurred (e.g., *Charletonia baluchestanica* and *Charletonia krendowskyi* on *Acrotylus insubricus*), they also selected different sites, i.e. hindwing and tympanum, respectively. With *Chorthippus* (*Chorthippus*) *dorsatus* as host, *Eutrombidium sorbasiensis* and *Eutrombidium* cf. *trigonum* simultaneously parasitized the hindwing and mesothorax, respectively. These patterns reinforce the notion that even closely related species partition microhabitats to reduce competition.

### Host specialization

Bipartite network analysis indicated a moderate level of specialization (H₂′ = 0.376), suggesting that the observed host–parasite associations are neither highly specialized nor completely generalized. Network connectivity was 0.21, meaning that about 21% of all possible host–parasite links were observed in the dataset. This relatively low connectivity suggests that many mite species interact with only a subset of the available hosts in the sampled community. Consistent with species-level specialization values (d′), *Eutrombidium sorbasiensis* showed the broadest host associations in the dataset, whereas several mite species were recorded from only a few hosts. However, these patterns should be interpreted cautiously, as limited associations may partly reflect sampling frequency rather than strict host restriction. Furthermore, the generality of parasites was 9.08, showing that on average, each mite species parasitized multiple host species, consistent with a generalist strategy. In contrast, the host vulnerability was relatively low (1.74), indicating that individual grasshopper species were typically infested by only a few mite taxa. These results together suggest that while mites exhibit broad host use, infestation pressure is not uniformly distributed across grasshopper species, pointing toward selective or ecologically constrained associations.

Some mite species, such as *Eutrombidium parishanensis*, *Leptus* (*Leptus*) *mallahae*, and *Parawenhoekia aginapaica*, were recorded only on few host species. Such species reduce network connectivity by increasing the number of potential links while contributing relatively few observed associations. Although this pattern may suggest a degree of host specificity, it should be interpreted cautiously. Differences in the phenology of mite species and their grasshopper hosts may influence the timing of host–parasite encounters, and interactions occurring outside the sampling time could therefore remain undetected. In addition, the relatively low sampling frequency and the low abundance of some mite species may further limit the detection of rare associations. Consequently, the apparent host restriction observed here may partly reflect incomplete sampling rather than strict ecological specialization.

## Conclusions

In this study, we investigated the host range, parasitological indices and host specificity of Parasitengona mites parasitizing Acrididae grasshoppers. By employing a network-based approach to analyze host-parasite interactions, we found that the overall bipartite network structure indicates a generalist parasitic strategy. However, variation in generality and vulnerability suggest that infestation pressure is unevenly distributed among grasshopper species, implying selective or ecologically constrained associations. Notably, our findings also acknowledge the potential influence of temporal sampling design, as the phenology of certain Parasitengona larvae may limit their occurrence to narrow seasonal windows. Furthermore, we identified specific host preferences regarding attachment sites and habitat associations. While these patterns are informative, the taxonomic uncertainty surrounding certain taxa, such as *Eutrombidium cf. trigonum* and specific immature grasshopper specimens, highlights the need for future molecular analyses to resolve these ambiguities and refine our understanding of host–parasite interactions. Our findings underscore the ecological significance of parasitic mites in shaping community dynamics and maintaining ecosystem health and offer valuable insights into the complexity of host-parasite interactions within ecological systems and reinforce the importance of continued investigation in this field. A deeper understanding of these dynamics is essential not only for advancing parasitological knowledge but also for informing biodiversity conservation efforts. In particular, elucidating host-parasite relationships can aid in identifying endemic species and designing targeted conservation strategies. Ultimately, this study highlights the critical role of parasitism in shaping ecological relationships and maintaining ecosystem health in the face of environmental change and habitat destruction.

## Supplementary Information

Below is the link to the electronic supplementary material.


Supplementary Material 1



Supplementary Material 2


## Data Availability

The mite specimens and grasshopper hosts were deposited in the Zoological Museum, College of Science, University of Tehran, Iran.
