# Supplementary material for: Host-parasite interactions between Acrididae (Orthoptera: Caelifera) and Parasitengona (Acari: Trombidiformes) in the southwestern Zagros Mountains, Iran
Source: Sci Rep. 2026 Jun 14;16:27151. doi: 10.1038/s41598-026-56417-5 (PMC13526880; doi:10.1038/s41598-026-56417-5)
Supplement: Supplementary file 1 [file 41598_2026_56417_MOESM1_ESM.docx]

**Host-parasite interactions between Acrididae (Orthoptera: Caelifera)** **and Parasitengona (Acari: Trombidiformes) in the southwestern** **Zagros** **Mountains, Iran**

Najmeh Kiany^1^, Marjan Seiedy^1^*, Masoud Hakimitabar^2^, Mohsen Kiany^3^ and Martin Husemann^4^

Supplementary Table 1. Mites’ attachment sites in grasshopper hosts whose pairwise comparisons were statistically significant (P *<* 0.05).

| **grasshopper species** | **Attachment sites** | **mite species** | **Adj. Sig.** | **grasshopper species** | **Attachment sites** | **mite species** | **Adj. Sig.** |
| --- | --- | --- | --- | --- | --- | --- | --- |
| ***O. miniata*** | **hindwing end-hindwing base** | ***E. sorbasiensis*** | **0.000** | ***N. anatolicus*** | **mesothorax-hindwing base** | ***E. sorbasiensis*** | **0.000** |
|  | **metathorax-hindwing base** |  | **0.000** |  | **abdomen-hindwing base** |  | **0.000** |
|  | **femur-hindwing base** |  | **0.000** |  | **coxa-hindwing base** |  | **0.000** |
|  | **tarsus-hindwing base** |  | **0.000** | ***R. bolivari*** | **hindwing end-hindwing base** |  | **0.009** |
|  | **head-hindwing base** |  | **0.000** |  | **metathorax-hindwing base** |  | **0.009** |
|  | **tympanum-hindwing base** |  | **0.000** |  | **femur-hindwing base** |  | **0.009** |
|  | **pronotum-hindwing base** |  | **0.000** |  | **tarsus-hindwing base** |  | **0.009** |
|  | **sternum-hindwing base** |  | **0.000** |  | **head-hindwing base** |  | **0.009** |
|  | **tegmina-hindwing base** |  | **0.000** |  | **tibia-hindwing base** |  | **0.009** |
|  | **mesothorax-hindwing base** |  | **0.000** |  | **tympanum-hindwing base** |  | **0.009** |
|  | **abdomen-hindwing base** |  | **0.000** |  | **pronotum-hindwing base** |  | **0.009** |
|  | **coxa-hindwing base** |  | **0.000** |  | **sternum-hindwing base** |  | **0.009** |
| ***M. wagneri*** | **hindwing end-hindwing base** |  | **0.004** |  | **tegmina-hindwing base** |  | **0.009** |
|  | **metathorax-hindwing base** |  | **0.004** |  | **mesothorax-hindwing base** |  | **0.009** |
|  | **femur-hindwing base** |  | **0.004** |  | **abdomen-hindwing base** |  | **0.009** |
|  | **tarsus-hindwing base** |  | **0.004** |  | **coxa-hindwing base** |  | **0.009** |
|  | **head-hindwing base** |  | **0.004** | ***S. xanthus*** | **hindwing end-hindwing base** |  | **0.000** |
|  | **tibia-hindwing base** |  | **0.004** |  | **metathorax-hindwing base** |  | **0.000** |
|  | **tympanum-hindwing base** |  | **0.004** |  | **femur-hindwing base** |  | **0.000** |
|  | **pronotum-hindwing base** |  | **0.004** |  | **tarsus-hindwing base** |  | **0.000** |
|  | **sternum-hindwing base** |  | **0.004** |  | **head-hindwing base** |  | **0.000** |
|  | **tegmina-hindwing base** |  | **0.004** |  | **tibia-hindwing base** |  | **0.000** |
|  | **mesothorax-hindwing base** |  | **0.004** |  | **tympanum-hindwing base** |  | **0.000** |
|  | **coxa-hindwing base** |  | **0.004** |  | **pronotum-hindwing base** |  | **0.000** |
| ***A. insubricus*** | **hindwing end-hindwing base** |  | **0.000** |  | **sternum-hindwing base** |  | **0.000** |
|  | **metathorax-hindwing base** |  | **0.000** |  | **tegmina-hindwing base** |  | **0.000** |
|  | **femur-hindwing base** |  | **0.000** |  | **mesothorax-hindwing base** |  | **0.000** |
|  | **tarsus-hindwing base** |  | **0.000** |  | **abdomen-hindwing base** |  | **0.000** |
|  | **head-hindwing base** |  | **0.000** |  | **coxa-hindwing base** |  | **0.000** |
|  | **tibia-hindwing base** |  | **0.000** | ***Notostaurus* sp.** | **hindwing end-hindwing base** |  | **0.011** |
|  | **tympanum-hindwing base** |  | **0.000** |  | **wing pad-hindwing base** |  | **0.011** |
|  | **pronotum-hindwing base** |  | **0.000** |  | **metathorax-hindwing base** |  | **0.011** |
|  | **sternum-hindwing base** |  | **0.000** |  | **femur-hindwing base** |  | **0.011** |
|  | **tegmina-hindwing base** |  | **0.000** |  | **tarsus-hindwing base** |  | **0.011** |
|  | **mesothorax-hindwing base** |  | **0.000** |  | **head-hindwing base** |  | **0.011** |
|  | **abdomen-hindwing base** |  | **0.000** |  | **tibia-hindwing base** |  | **0.011** |
|  | **coxa-hindwing base** |  | **0.000** |  | **tympanum-hindwing base** |  | **0.011** |
| ***S. theodori*** | **hindwing end-hindwing base** |  | **0.000** |  | **pronotum-hindwing base** |  | **0.011** |
|  | **metathorax-hindwing base** |  | **0.000** |  | **sternum-hindwing base** |  | **0.011** |
|  | **femur-hindwing base** |  | **0.000** |  | **tegmina-hindwing base** |  | **0.011** |
|  | **tarsus-hindwing base** |  | **0.000** |  | **mesothorax-hindwing base** |  | **0.011** |
|  | **head-hindwing base** |  | **0.000** |  | **abdomen-hindwing base** |  | **0.011** |
|  | **tibia-hindwing base** |  | **0.000** |  | **coxa-hindwing base** |  | **0.011** |
|  | **tympanum-hindwing base** |  | **0.000** | ***A. thalassinus*** | **hindwing end-hindwing base** |  | **0.000** |
|  | **pronotum-hindwing base** |  | **0.000** |  | **metathorax-hindwing base** |  | **0.000** |
|  | **sternum-hindwing base** |  | **0.000** |  | **femur-hindwing base** |  | **0.000** |
|  | **tegmina-hindwing base** |  | **0.000** |  | **tarsus-hindwing base** |  | **0.000** |
|  | **mesothorax-hindwing base** |  | **0.000** |  | **head-hindwing base** |  | **0.000** |
|  | **abdomen-hindwing base** |  | **0.000** |  | **tibia-hindwing base** |  | **0.000** |
|  | **coxa-hindwing base** |  | **0.000** |  | **tympanum-hindwing base** |  | **0.000** |
| ***S. rubescens*** | **hindwing end-hindwing base** |  | **0.000** |  | **pronotum-hindwing base** |  | **0.000** |
|  | **metathorax-hindwing base** |  | **0.000** |  | **sternum-hindwing base** |  | **0.000** |
|  | **femur-hindwing base** |  | **0.000** |  | **tegmina-hindwing base** |  | **0.000** |
|  | **tarsus-hindwing base** |  | **0.000** |  | **mesothorax-hindwing base** |  | **0.000** |
|  | **head-hindwing base** |  | **0.000** |  | **abdomen-hindwing base** |  | **0.000** |
|  | **tibia-hindwing base** |  | **0.000** |  | **coxa-hindwing base** |  | **0.000** |
|  | **tympanum-hindwing base** |  | **0.000** |  | **hindwing base-tibia** |  | **0.027** |
|  | **pronotum-hindwing base** |  | **0.000** | ***C. barbarus*** | **hindwing end-tibia** | ***L. darvishi*** | **0.027** |
|  | **sternum-hindwing base** |  | **0.000** |  | **tegmina-tibia** |  | **0.027** |
|  | **tegmina-hindwing base** |  | **0.000** |  | **pronotum-tibia** |  | **0.027** |
|  | **mesothorax-hindwing base** |  | **0.000** |  | **sternum-tibia** |  | **0.027** |
|  | **abdomen-hindwing base** |  | **0.000** |  | **tympanum-tibia** |  | **0.027** |
|  | **coxa-hindwing base** |  | **0.000** |  | **coxa-tibia** |  | **0.027** |
| ***S. coerulipes*** | **hindwing end-hindwing base** |  | **0.000** |  | **head-tibia** |  | **0.027** |
|  | **metathorax-hindwing base** |  | **0.000** |  | **mesothorax-tibia** |  | **0.027** |
|  | **femur-hindwing base** |  | **0.000** |  | **hindwing end-tibia** |  | **0.017** |
|  | **tarsus-hindwing base** |  | **0.000** | ***N. anatolicus*** | **tegmina-tibia** |  | **0.017** |
|  | **head-hindwing base** |  | **0.000** |  | **tympanum-tibia** |  | **0.017** |
|  | **tibia-hindwing base** |  | **0.000** |  | **abdomen-tibia** |  | **0.017** |
|  | **tympanum-hindwing base** |  | **0.000** |  | **coxa-tibia** |  | **0.017** |
|  | **pronotum-hindwing base** |  | **0.000** |  | **metathorax-tibia** |  | **0.017** |
|  | **sternum-hindwing base** |  | **0.000** |  | **head-hindwing base** |  | **0.004** |
|  | **tegmina-hindwing base** |  | **0.000** |  | **tegmina-hindwing base** |  | **0.004** |
|  | **mesothorax-hindwing base** |  | **0.000** | ***O. miniata*** | **pronotum-hindwing base** | ***C. baluchestanica*** | **0.004** |
|  | **abdomen-hindwing base** |  | **0.000** |  | **mesothorax-hindwing base** |  | **0.004** |
|  | **coxa-hindwing base** |  | **0.000** |  | **metathorax-hindwing base** |  | **0.004** |
| ***S. nebulosus persa*** | **hindwing end-hindwing base** |  | **0.000** |  | **sternum-hindwing base** |  | **0.004** |
|  | **metathorax-hindwing base** |  | **0.000** |  | **tympanum-hindwing base** |  | **0.004** |
|  | **femur-hindwing base** |  | **0.000** |  | **abdomen-hindwing base** |  | **0.004** |
|  | **tarsus-hindwing base** |  | **0.000** |  | **coxa-hindwing base** |  | **0.004** |
|  | **head-hindwing base** |  | **0.000** |  | **femur-hindwing base** |  | **0.004** |
|  | **tibia-hindwing base** |  | **0.000** |  | **tibia-hindwing base** |  | **0.004** |
|  | **tympanum-hindwing base** |  | **0.000** |  | **tarsus-hindwing base** |  | **0.004** |
|  | **pronotum-hindwing base** |  | **0.000** |  | **head-hindwing base** |  | **0.001** |
|  | **sternum-hindwing base** |  | **0.000** |  | **tegmina-hindwing base** |  | **0.024** |
|  | **tegmina-hindwing base** |  | **0.000** | ***A. insubricus*** | **pronotum-hindwing base** |  | **0.001** |
|  | **metathorax-hindwing base** |  | **0.000** |  | **mesothorax-hindwing base** |  | **0.001** |
|  | **femur-hindwing base** |  | **0.000** |  | **metathorax-hindwing base** |  | **0.001** |
|  | **tarsus-hindwing base** |  | **0.000** |  | **sternum-hindwing base** |  | **0.024** |
|  | **head-hindwing base** |  | **0.000** |  | **tympanum-hindwing base** |  | **0.001** |
|  | **tibia-hindwing base** |  | **0.000** |  | **abdomen-hindwing base** |  | **0.024** |
|  | **tympanum-hindwing base** |  | **0.000** |  | **coxa-hindwing base** |  | **0.001** |
|  | **pronotum-hindwing base** |  | **0.000** |  | **tibia-hindwing base** |  | **0.024** |
|  | **sternum-hindwing base** |  | **0.000** |  | **tarsus-hindwing base** |  | **0.001** |
|  | **tegmina-hindwing base** |  | **0.000** |  | **head-hindwing base** |  | **0.011** |
|  | **mesothorax-hindwing base** |  | **0.000** |  | **tegmina-hindwing base** |  | **0.011** |
|  | **abdomen-hindwing base** |  | **0.000** | ***T. mesopotamica*** | **pronotum-hindwing base** |  | **0.011** |
|  | **coxa-hindwing base** |  | **0.000** |  | **mesothorax-hindwing base** |  | **0.011** |
| ***S. carinatus*** | **hindwing end-hindwing base** |  | **0.000** |  | **metathorax-hindwing base** |  | **0.011** |
|  | **metathorax-hindwing base** |  | **0.000** |  | **sternum-hindwing base** |  | **0.011** |
|  | **femur-hindwing base** |  | **0.000** |  | **tympanum-hindwing base** |  | **0.011** |
|  | **tarsus-hindwing base** |  | **0.000** |  | **abdomen-hindwing base** |  | **0.011** |
|  | **head-hindwing base** |  | **0.000** |  | **coxa-hindwing base** |  | **0.011** |
|  | **tibia-hindwing base** |  | **0.000** |  | **femur-hindwing base** |  | **0.011** |
|  | **tympanum-hindwing base** |  | **0.000** |  | **tibia-hindwing base** |  | **0.011** |
|  | **pronotum-hindwing base** |  | **0.000** |  | **tarsus-hindwing base** |  | **0.011** |
|  | **sternum-hindwing base** |  | **0.000** |  | **hindwing end-hindwing base** |  | **0.011** |
|  | **tegmina-hindwing base** |  | **0.000** |  | **hindwing end-hindwing base** |  | **0.000** |
|  | **mesothorax-hindwing base** |  | **0.000** |  | **tegmina-hindwing base** |  | **0.000** |
| ***S. angustus*** | **hindwing end-hindwing base** |  | **0.011** | ***A. insubricus*** | **pronotum-hindwing base** | ***Eutrombidium* sp. nov.** | **0.000** |
|  | **metathorax-hindwing base** |  | **0.011** |  | **mesothorax-hindwing base** |  | **0.000** |
|  | **femur-hindwing base** |  | **0.011** |  | **metathorax-hindwing base** |  | **0.000** |
|  | **tarsus-hindwing base** |  | **0.011** |  | **sternum-hindwing base** |  | **0.000** |
|  | **head-hindwing base** |  | **0.011** |  | **tympanum-hindwing base** |  | **0.000** |
|  | **tibia-hindwing base** |  | **0.011** |  | **abdomen-hindwing base** |  | **0.000** |
|  | **pronotum-hindwing base** |  | **0.011** |  | **coxa-hindwing base** |  | **0.000** |
|  | **sternum-hindwing base** |  | **0.011** |  | **femur-hindwing base** |  | **0.000** |
|  | **tegmina-hindwing base** |  | **0.011** |  | **tibia-hindwing base** |  | **0.000** |
|  | **abdomen-hindwing base** |  | **0.011** |  | **tarsus-hindwing base** |  | **0.000** |
|  | **coxa-hindwing base** |  | **0.011** |  | **head-hindwing base** |  | **0.000** |
| ***A. simulatrix*** | **hindwing end-hindwing base** |  | **0.01** |  | **hindwing end-hindwing base** |  | **0.011** |
|  | **metathorax-hindwing base** |  | **0.01** |  | **tegmina-hindwing base** |  | **0.011** |
|  | **femur-hindwing base** |  | **0.01** | ***S. rubescens*** | **pronotum-hindwing base** |  | **0.011** |
|  | **tarsus-hindwing base** |  | **0.01** |  | **mesothorax-hindwing base** |  | **0.011** |
|  | **head-hindwing base** |  | **0.01** |  | **metathorax-hindwing base** |  | **0.011** |
|  | **tympanum-hindwing base** |  | **0.01** |  | **sternum-hindwing base** |  | **0.011** |
|  | **pronotum-hindwing base** |  | **0.01** |  | **tympanum-hindwing base** |  | **0.011** |
|  | **sternum-hindwing base** |  | **0.01** |  | **abdomen-hindwing base** |  | **0.011** |
|  | **tegmina-hindwing base** |  | **0.01** |  | **coxa-hindwing base** |  | **0.011** |
|  | **mesothorax-hindwing base** |  | **0.01** |  | **femur-hindwing base** |  | **0.011** |
|  | **abdomen-hindwing base** |  | **0.01** |  | **tibia-hindwing base** |  | **0.011** |
|  | **coxa-hindwing base** |  | **0.01** |  | **tarsus-hindwing base** |  | **0.011** |
| ***Aiolopus* sp.** | **hindwing end-hindwing base** |  | **0.000** |  | **head-hindwing base** |  | **0.011** |
|  | **metathorax-hindwing base** |  | **0.000** |  | **hindwing end-hindwing base** |  | **0.000** |
|  | **femur-hindwing base** |  | **0.000** |  | **tegmina-hindwing base** |  | **0.000** |
|  | **tarsus-hindwing base** |  | **0.000** | ***C. barbarus*** | **pronotum-hindwing base** |  | **0.000** |
|  | **head-hindwing base** |  | **0.000** |  | **mesothorax-hindwing base** |  | **0.000** |
|  | **tegmina-hindwing base** |  | **0.000** |  | **metathorax-hindwing base** |  | **0.000** |
|  | **mesothorax-hindwing base** |  | **0.000** |  | **sternum-hindwing base** |  | **0.000** |
|  | **abdomen-hindwing base** |  | **0.000** |  | **tympanum-hindwing base** |  | **0.000** |
|  | **coxa-hindwing base** |  | **0.000** |  | **abdomen-hindwing base** |  | **0.000** |
| ***O. kurda*** | **hindwing end-hindwing base** |  | **0.000** |  | **coxa-hindwing base** |  | **0.000** |
|  | **metathorax-hindwing base** |  | **0.000** |  | **femur-hindwing base** |  | **0.000** |
|  | **femur-hindwing base** |  | **0.000** |  | **tibia-hindwing base** |  | **0.000** |
|  | **tarsus-hindwing base** |  | **0.000** |  | **tarsus-hindwing base** |  | **0.000** |
|  | **head-hindwing base** |  | **0.000** |  | **head-hindwing base** |  | **0.000** |
|  | **tibia-hindwing base** |  | **0.000** |  | **hindwing end-hindwing base** |  | **0.000** |
|  | **tympanum-hindwing base** |  | **0.000** |  | **tegmina-hindwing base** |  | **0.001** |
|  | **pronotum-hindwing base** |  | **0.000** | ***Ch. dorsatus*** | **pronotum-hindwing base** |  | **0.000** |
|  | **sternum-hindwing base** |  | **0.000** |  | **mesothorax-hindwing base** |  | **0.001** |
|  | **tegmina-hindwing base** |  | **0.000** |  | **metathorax-hindwing base** |  | **0.000** |
|  | **mesothorax-hindwing base** |  | **0.000** |  | **sternum-hindwing base** |  | **0.000** |
|  | **abdomen-hindwing base** |  | **0.000** |  | **tympanum-hindwing base** |  | **0.002** |
|  | **coxa-hindwing base** |  | **0.000** |  | **abdomen-hindwing base** |  | **0.002** |
| ***P. armata*** | **hindwing end-hindwing base** |  | **0.000** |  | **coxa-hindwing base** |  | **0.000** |
|  | **metathorax-hindwing base** |  | **0.000** |  | **femur-hindwing base** |  | **0.000** |
|  | **tibia-hindwing base** |  | **0.000** |  | **tibia-hindwing base** |  | **0.000** |
|  | **tympanum-hindwing base** |  | **0.000** |  | **tarsus-hindwing base** |  | **0.000** |
|  | **pronotum-hindwing base** |  | **0.000** |  | **head-hindwing base** |  | **0.000** |
|  | **sternum-hindwing base** |  | **0.000** |  | **hindwing end-hindwing base** |  | **0.000** |
|  | **tegmina-hindwing base** |  | **0.000** |  | **tegmina-hindwing base** |  | **0.000** |
|  | **mesothorax-hindwing base** |  | **0.000** | ***Chorthippus* sp*.*** | **pronotum-hindwing base** |  | **0.000** |
|  | **abdomen-hindwing base** |  | **0.000** |  | **mesothorax-hindwing base** |  | **0.000** |
|  | **coxa-hindwing base** |  | **0.000** |  | **metathorax-hindwing base** |  | **0.000** |
| ***Ps. dirshi*** | **hindwing end-hindwing base** |  | **0.000** |  | **sternum-hindwing base** |  | **0.000** |
|  | **metathorax-hindwing base** |  | **0.000** |  | **tympanum-hindwing base** |  | **0.000** |
|  | **femur-hindwing base** |  | **0.000** |  | **abdomen-hindwing base** |  | **0.000** |
|  | **tarsus-hindwing base** |  | **0.000** |  | **coxa-hindwing base** |  | **0.000** |
|  | **head-hindwing base** |  | **0.000** |  | **femur-hindwing base** |  | **0.000** |
|  | **tibia-hindwing base** |  | **0.000** |  | **tibia-hindwing base** |  | **0.000** |
|  | **tympanum-hindwing base** |  | **0.000** |  | **tarsus-hindwing base** |  | **0.000** |
|  | **pronotum-hindwing base** |  | **0.000** |  | **head-hindwing base** |  | **0.000** |
|  | **sternum-hindwing base** |  | **0.000** |  | **hindwing end-hindwing base** |  | **0.000** |
|  | **tegmina-hindwing base** |  | **0.000** |  | **tegmina-hindwing base** |  | **0.000** |
|  | **mesothorax-hindwing base** |  | **0.000** | ***O. miniata*** | **pronotum-hindwing base** | ***C. krendowskyi*** | **0.000** |
|  | **abdomen-hindwing base** |  | **0.000** |  | **mesothorax-hindwing base** |  | **0.000** |
|  | **coxa-hindwing base** |  | **0.000** |  | **metathorax-hindwing base** |  | **0.000** |
| ***C. barbarus*** | **hindwing end-hindwing base** |  | **0.000** |  | **sternum-hindwing base** |  | **0.000** |
|  | **metathorax-hindwing base** |  | **0.000** |  | **tympanum-hindwing base** |  | **0.000** |
|  | **femur-hindwing base** |  | **0.000** |  | **abdomen-hindwing base** |  | **0.000** |
|  | **tarsus-hindwing base** |  | **0.000** |  | **coxa-hindwing base** |  | **0.000** |
|  | **head-hindwing base** |  | **0.000** |  | **femur-hindwing base** |  | **0.000** |
|  | **tibia-hindwing base** |  | **0.000** |  | **tibia-hindwing base** |  | **0.000** |
|  | **tympanum-hindwing base** |  | **0.000** |  | **tarsus-hindwing base** |  | **0.000** |
|  | **pronotum-hindwing base** |  | **0.000** |  | **head-hindwing base** |  | **0.000** |
|  | **sternum-hindwing base** |  | **0.000** |  | **tegmina-hindwing base** |  | **0.022** |
|  | **tegmina-hindwing base** |  | **0.000** |  | **pronotum-hindwing base** |  | **0.022** |
|  | **mesothorax-hindwing base** |  | **0.000** | ***T. mesopotamica*** | **metathorax-hindwing base** |  | **0.022** |
|  | **abdomen-hindwing base** |  | **0.000** |  | **sternum-hindwing base** |  | **0.022** |
|  | **coxa-hindwing base** |  | **0.000** |  | **tympanum-hindwing base** |  | **0.022** |
| ***C. tenuicercis*** | **hindwing end-hindwing base** |  | **0.000** |  | **abdomen-hindwing base** |  | **0.022** |
|  | **metathorax-hindwing base** |  | **0.000** |  | **coxa-hindwing base** |  | **0.022** |
|  | **femur-hindwing base** |  | **0.000** |  | **femur-hindwing base** |  | **0.022** |
|  | **tarsus-hindwing base** |  | **0.000** |  | **tibia-hindwing base** |  | **0.022** |
|  | **head-hindwing base** |  | **0.000** |  | **tarsus-hindwing base** |  | **0.022** |
|  | **tibia-hindwing base** |  | **0.000** |  | **head-hindwing base** |  | **0.022** |
|  | **tympanum-hindwing base** |  | **0.000** |  | **hindwing end-hindwing base** |  | **0.011** |
|  | **pronotum-hindwing base** |  | **0.000** |  | **tegmina-hindwing base** |  | **0.011** |
|  | **sternum-hindwing base** |  | **0.000** |  | **pronotum-hindwing base** |  | **0.011** |
|  | **mesothorax-hindwing base** |  | **0.000** | ***A. aegyptium*** | **mesothorax-hindwing base** |  | **0.011** |
|  | **abdomen-hindwing base** |  | **0.000** |  | **metathorax-hindwing base** |  | **0.011** |
|  | **coxa-hindwing base** |  | **0.000** |  | **sternum-hindwing base** |  | **0.011** |
| ***C. coelesyriensis*** | **hindwing end-hindwing base** |  | **0.000** |  | **tympanum-hindwing base** |  | **0.011** |
|  | **femur-hindwing base** |  | **0.000** |  | **abdomen-hindwing base** |  | **0.011** |
|  | **tarsus-hindwing base** |  | **0.000** |  | **coxa-hindwing base** |  | **0.011** |
|  | **head-hindwing base** |  | **0.000** |  | **femur-hindwing base** |  | **0.011** |
|  | **tibia-hindwing base** |  | **0.000** |  | **tibia-hindwing base** |  | **0.011** |
|  | **tympanum-hindwing base** |  | **0.000** |  | **tarsus-hindwing base** |  | **0.011** |
|  | **pronotum-hindwing base** |  | **0.000** |  | **head-hindwing base** |  | **0.011** |
|  | **sternum-hindwing base** |  | **0.000** |  | **hindwing end-hindwing base** |  | **0.000** |
|  | **tegmina-hindwing base** |  | **0.000** |  | **tegmina-hindwing base** |  | **0.000** |
|  | **mesothorax-hindwing base** |  | **0.000** |  | **pronotum-hindwing base** |  | **0.000** |
|  | **abdomen-hindwing base** |  | **0.000** | ***Ch. dorsatus*** | **mesothorax-hindwing base** |  | **0.000** |
|  | **coxa-hindwing base** |  | **0.000** |  | **metathorax-hindwing base** |  | **0.000** |
| ***S. luteipes*** | **hindwing end-hindwing base** |  | **0.000** |  | **sternum-hindwing base** |  | **0.000** |
|  | **metathorax-hindwing base** |  | **0.000** |  | **tympanum-hindwing base** |  | **0.000** |
|  | **femur-hindwing base** |  | **0.000** |  | **abdomen-hindwing base** |  | **0.000** |
|  | **tarsus-hindwing base** |  | **0.000** |  | **coxa-hindwing base** |  | **0.000** |
|  | **head-hindwing base** |  | **0.000** |  | **femur-hindwing base** |  | **0.000** |
|  | **tibia-hindwing base** |  | **0.000** |  | **tibia-hindwing base** |  | **0.000** |
|  | **tympanum-hindwing base** |  | **0.000** |  | **tarsus-hindwing base** |  | **0.000** |
|  | **pronotum-hindwing base** |  | **0.000** |  | **head-hindwing base** |  | **0.000** |
|  | **sternum-hindwing base** |  | **0.000** |  | **hindwing end-metathorax** |  | **0.000** |
|  | **tegmina-hindwing base** |  | **0.000** |  | **tegmina-metathorax** |  | **0.000** |
|  | **mesothorax-hindwing base** |  | **0.000** |  | **mesothorax-metathorax** |  | **0.000** |
|  | **abdomen-hindwing base** |  | **0.000** | ***M. wagneri*** | **sternum-metathorax** | ***C. ahwazensis*** | **0.000** |
| ***H. persa*** | **hindwing end-hindwing base** |  | **0.000** |  | **tympanum-metathorax** |  | **0.000** |
|  | **metathorax-hindwing base** |  | **0.000** |  | **tibia-metathorax** |  | **0.000** |
|  | **femur-hindwing base** |  | **0.000** |  | **tarsus-metathorax** |  | **0.000** |
|  | **tarsus-hindwing base** |  | **0.000** |  | **head-metathorax** |  | **0.000** |
|  | **head-hindwing base** |  | **0.000** |  | **femur-metathorax** |  | **0.000** |
|  | **tibia-hindwing base** |  | **0.000** |  | **pronotum-metathorax** |  | **0.002** |
|  | **tympanum-hindwing base** |  | **0.000** |  | **coxa-metathorax** |  | **0.021** |
|  | **pronotum-hindwing base** |  | **0.000** |  | **hindwing end-metathorax** |  | **0.000** |
|  | **sternum-hindwing base** |  | **0.000** |  | **tegmina-metathorax** |  | **0.000** |
|  | **tegmina-hindwing base** |  | **0.000** |  | **mesothorax-metathorax** |  | **0.000** |
|  | **mesothorax-hindwing base** |  | **0.000** | ***H. adspersa*** | **sternum-metathorax** |  | **0.000** |
|  | **abdomen-hindwing base** |  | **0.000** |  | **tympanum-metathorax** |  | **0.000** |
|  | **coxa-hindwing base** |  | **0.000** |  | **tibia-metathorax** |  | **0.000** |
| ***H. pterosticha*** | **hindwing end-hindwing base** |  | **0.011** |  | **tarsus-metathorax** |  | **0.000** |
|  | **metathorax-hindwing base** |  | **0.011** |  | **head-metathorax** |  | **0.000** |
|  | **femur-hindwing base** |  | **0.011** |  | **femur-metathorax** |  | **0.000** |
|  | **tarsus-hindwing base** |  | **0.011** |  | **pronotum-metathorax** |  | **0.000** |
|  | **head-hindwing base** |  | **0.011** |  | **coxa-metathorax** |  | **0.000** |
|  | **tibia-hindwing base** |  | **0.011** |  | **abdomen-metathorax** |  | **0.000** |
|  | **tympanum-hindwing base** |  | **0.011** |  | **hindwing base-metathorax** |  | **0.000** |
|  | **pronotum-hindwing base** |  | **0.011** |  | **hindwing base-tarsus** |  | **0.004** |
|  | **sternum-hindwing base** |  | **0.011** |  | **hindwing end-tarsus** |  | **0.004** |
|  | **tegmina-hindwing base** |  | **0.011** |  | **tegmina-tarsus** |  | **0.004** |
|  | **mesothorax-hindwing base** |  | **0.011** | ***Notostaurus anatolicus*** | **pronotum-tarsus** | ***T. hessabyi*** | **0.004** |
|  | **abdomen-hindwing base** |  | **0.011** |  | **mesothorax-tarsus** |  | **0.004** |
|  | **coxa-hindwing base** |  | **0.011** |  | **metathorax-tarsus** |  | **0.004** |
| ***A. oxycephala*** | **hindwing end-hindwing base** |  | **0.000** |  | **sternum-tarsus** |  | **0.004** |
|  | **metathorax-hindwing base** |  | **0.000** |  | **tympanum-tarsus** |  | **0.004** |
|  | **femur-hindwing base** |  | **0.000** |  | **coxa-tarsus** |  | **0.004** |
|  | **tarsus-hindwing base** |  | **0.000** |  | **femur-tarsus** |  | **0.004** |
|  | **head-hindwing base** |  | **0.000** |  | **tibia-tarsus** |  | **0.004** |
|  | **tibia-hindwing base** |  | **0.000** |  | **head-tarsus** |  | **0.004** |
|  | **tympanum-hindwing base** |  | **0.000** |  | **hindwing base-tarsus** |  | **0.000** |
|  | **pronotum-hindwing base** |  | **0.000** |  | **hindwing end-tarsus** |  | **0.000** |
|  | **sternum-hindwing base** |  | **0.000** |  | **tegmina-tarsus** |  | **0.000** |
|  | **mesothorax-hindwing base** |  | **0.000** | ***Notostaurus* sp.** | **wing pad-tarsus** |  | **0.000** |
|  | **abdomen-hindwing base** |  | **0.000** |  | **pronotum-tarsus** |  | **0.000** |
|  | **coxa-hindwing base** |  | **0.000** |  | **mesothorax-tarsus** |  | **0.000** |
| ***T. mesopotamica*** | **hindwing end-hindwing base** |  | **0.000** |  | **metathorax-tarsus** |  | **0.000** |
|  | **femur-hindwing base** |  | **0.000** |  | **sternum-tarsus** |  | **0.000** |
|  | **tarsus-hindwing base** |  | **0.000** |  | **tympanum-tarsus** |  | **0.000** |
|  | **head-hindwing base** |  | **0.000** |  | **coxa-tarsus** |  | **0.000** |
|  | **tibia-hindwing base** |  | **0.000** |  | **femur-tarsus** |  | **0.000** |
|  | **pronotum-hindwing base** |  | **0.000** |  | **tibia-tarsus** |  | **0.000** |
|  | **sternum-hindwing base** |  | **0.000** |  | **head-tarsus** |  | **0.000** |
|  | **tegmina-hindwing base** |  | **0.000** |  | **abdomen-tarsus** |  | **0.000** |
|  | **mesothorax-hindwing base** |  | **0.000** |  | **tympanum-hindwing base** |  | **0.004** |
|  | **coxa-hindwing base** |  | **0.000** |  | **tarsus-hindwing base** |  | **0.004** |
| ***E. farsistanicus*** | **hindwing end-hindwing base** |  | **0.000** |  | **tympanum-femur** |  | **0.005** |
|  | **metathorax-hindwing base** |  | **0.000** | ***O. miniata*** | **tarsus-femur** | ***A. lorestanicus*** | **0.005** |
|  | **femur-hindwing base** |  | **0.000** |  | **hindwing end-femur** |  | **0.019** |
|  | **tarsus-hindwing base** |  | **0.000** |  | **tegmina-femur** |  | **0.019** |
|  | **head-hindwing base** |  | **0.000** |  | **hindwing end-hindwing base** |  | **0.018** |
|  | **tibia-hindwing base** |  | **0.000** |  | **tegmina-hindwing base** |  | **0.018** |
|  | **tympanum-hindwing base** |  | **0.000** |  | **abdomen-hindwing base** |  | **0.018** |
|  | **pronotum-hindwing base** |  | **0.000** |  | **abdomen-femur** |  | **0.019** |
|  | **sternum-hindwing base** |  | **0.000** |  | **tarsus-pronotum** |  | **0.011** |
|  | **mesothorax-hindwing base** |  | **0.000** |  | **tympanum-pronotum** |  | **0.011** |
|  | **coxa-hindwing base** |  | **0.000** |  | **tegmina-pronotum** |  | **0.011** |
| ***Ch. brunneus*** | **hindwing end-hindwing base** |  | **0.000** |  | **abdomen-pronotum** |  | **0.011** |
|  | **metathorax-hindwing base** |  | **0.000** | ***S. theodori*** | **hindwing end-pronotum** |  | **0.011** |
|  | **femur-hindwing base** |  | **0.000** |  | **sternum-pronotum** |  | **0.011** |
|  | **tarsus-hindwing base** |  | **0.000** |  | **head-pronotum** |  | **0.011** |
|  | **head-hindwing base** |  | **0.000** |  | **coxa-pronotum** |  | **0.011** |
|  | **tibia-hindwing base** |  | **0.000** |  | **metathorax-pronotum** |  | **0.011** |
|  | **tympanum-hindwing base** |  | **0.000** |  | **tibia-pronotum** |  | **0.011** |
|  | **pronotum-hindwing base** |  | **0.000** |  | **hindwing base-pronotum** |  | **0.011** |
|  | **sternum-hindwing base** |  | **0.000** |  | **tarsus-mesothorax** |  | **0.000** |
|  | **tegmina-hindwing base** |  | **0.000** |  | **tympanum-mesothorax** |  | **0.000** |
|  | **mesothorax-hindwing base** |  | **0.000** |  | **tegmina-mesothorax** |  | **0.000** |
|  | **abdomen-hindwing base** |  | **0.000** |  | **abdomen-mesothorax** |  | **0.000** |
|  | **coxa-hindwing base** |  | **0.000** | ***S. coerulipes*** | **hindwing end-mesothorax** |  | **0.000** |
| ***Ch. dorsatus*** | **hindwing end-hindwing base** |  | **0.000** |  | **sternum-mesothorax** |  | **0.000** |
|  | **metathorax-hindwing base** |  | **0.000** |  | **head-mesothorax** |  | **0.000** |
|  | **femur-hindwing base** |  | **0.000** |  | **coxa-mesothorax** |  | **0.000** |
|  | **tarsus-hindwing base** |  | **0.000** |  | **metathorax-mesothorax** |  | **0.000** |
|  | **head-hindwing base** |  | **0.000** |  | **tibia-mesothorax** |  | **0.000** |
|  | **tibia-hindwing base** |  | **0.000** |  | **hindwing base-mesothorax** |  | **0.000** |
|  | **tympanum-hindwing base** |  | **0.000** |  | **femur-mesothorax** |  | **0.000** |
|  | **pronotum-hindwing base** |  | **0.000** |  | **pronotum-mesothorax** |  | **0.024** |
|  | **sternum-hindwing base** |  | **0.000** |  | **tympanum-femur** |  | **0.027** |
|  | **tegmina-hindwing base** |  | **0.000** |  | **hindwing end-femur** |  | **0.027** |
|  | **mesothorax-hindwing base** |  | **0.000** |  | **tegmina-femur** |  | **0.027** |
|  | **abdomen-hindwing base** |  | **0.000** |  | **sternum-femur** |  | **0.027** |
|  | **coxa-hindwing base** |  | **0.000** | ***S. pilosus*** | **coxa-femur** |  | **0.027** |
| ***Chorthippus* sp.** | **hindwing end-hindwing base** |  | **0.000** |  | **metathorax-femur** |  | **0.027** |
|  | **metathorax-hindwing base** |  | **0.000** |  | **mesothorax-femur** |  | **0.027** |
|  | **femur-hindwing base** |  | **0.000** |  | **pronotum-femur** |  | **0.027** |
|  | **tarsus-hindwing base** |  | **0.000** |  | **hindwing base-femur** |  | **0.027** |
|  | **head-hindwing base** |  | **0.000** |  | **tarsus-pronotum** |  | **0.000** |
|  | **tibia-hindwing base** |  | **0.000** |  | **tympanum-pronotum** |  | **0.000** |
|  | **tympanum-hindwing base** |  | **0.000** |  | **tegmina-pronotum** |  | **0.000** |
|  | **pronotum-hindwing base** |  | **0.000** |  | **abdomen-pronotum** |  | **0.000** |
|  | **sternum-hindwing base** |  | **0.000** | ***O. kurda*** | **hindwing end-pronotum** |  | **0.000** |
|  | **tegmina-hindwing base** |  | **0.000** |  | **sternum-pronotum** |  | **0.000** |
|  | **mesothorax-hindwing base** |  | **0.000** |  | **head-pronotum** |  | **0.000** |
|  | **abdomen-hindwing base** |  | **0.000** |  | **coxa-pronotum** |  | **0.000** |
|  | **coxa-hindwing base** |  | **0.000** |  | **metathorax-pronotum** |  | **0.000** |
| ***D. tartarus*** | **hindwing end-hindwing base** |  | **0.000** |  | **tibia-pronotum** |  | **0.000** |
|  | **metathorax-hindwing base** |  | **0.000** |  | **mesothorax-pronotum** |  | **0.000** |
|  | **femur-hindwing base** |  | **0.000** |  | **hindwing base-pronotum** |  | **0.011** |
|  | **tarsus-hindwing base** |  | **0.000** |  | **femur-pronotum** |  | **0.011** |
|  | **head-hindwing base** |  | **0.000** |  | **tympanum-hindwing base** |  | **0.001** |
|  | **tibia-hindwing base** |  | **0.000** |  | **tarsus-hindwing base** |  | **0.001** |
|  | **tympanum-hindwing base** |  | **0.000** |  | **hindwing end-hindwing base** |  | **0.001** |
|  | **pronotum-hindwing base** |  | **0.000** |  | **abdomen-hindwing base** |  | **0.001** |
|  | **sternum-hindwing base** |  | **0.000** | ***N. anatolicus*** | **sternum-hindwing base** |  | **0.001** |
|  | **tegmina-hindwing base** |  | **0.000** |  | **head-hindwing base** |  | **0.001** |
|  | **mesothorax-hindwing base** |  | **0.000** |  | **coxa-hindwing base** |  | **0.001** |
|  | **abdomen-hindwing base** |  | **0.000** |  | **metathorax-hindwing base** |  | **0.001** |
|  | **coxa-hindwing base** |  | **0.000** |  | **tibia-hindwing base** |  | **0.001** |
| ***D. hauensteini farsistanicus*** | **hindwing end-hindwing base** |  | **0.000** |  | **mesothorax-hindwing base** |  | **0.001** |
|  | **metathorax-hindwing base** |  | **0.000** |  | **femur-hindwing base** |  | **0.001** |
|  | **femur-hindwing base** |  | **0.000** |  | **wing pad-tarsus** |  | **0.011** |
|  | **tarsus-hindwing base** |  | **0.000** |  | **tympanum-tarsus** |  | **0.011** |
|  | **head-hindwing base** |  | **0.000** |  | **hindwing base-tarsus** |  | **0.011** |
|  | **tibia-hindwing base** |  | **0.000** |  | **hindwing end-tarsus** |  | **0.011** |
|  | **tympanum-hindwing base** |  | **0.000** | ***Notostaurus* sp*.*** | **tegmina-tarsus** |  | **0.011** |
|  | **pronotum-hindwing base** |  | **0.000** |  | **metathorax-tarsus** |  | **0.011** |
|  | **sternum-hindwing base** |  | **0.000** |  | **sternum-tarsus** |  | **0.011** |
|  | **tegmina-hindwing base** |  | **0.000** |  | **abdomen-tarsus** |  | **0.011** |
|  | **mesothorax-hindwing base** |  | **0.000** |  | **coxa-tarsus** |  | **0.011** |
|  | **abdomen-hindwing base** |  | **0.000** |  | **tibia-tarsus** |  | **0.011** |
|  | **coxa-hindwing base** |  | **0.000** |  | **femur-tarsus** |  | **0.011** |
| ***N. anatolicus*** | **hindwing end-hindwing base** |  | **0.000** |  | **pronotum-tarsus** |  | **0.011** |
|  | **metathorax-hindwing base** |  | **0.000** |  | **mesothorax-tarsus** |  | **0.011** |
|  | **femur-hindwing base** |  | **0.000** |  | **head-tarsus** |  | **0.011** |
|  | **tarsus-hindwing base** |  | **0.000** |  |  |  |  |
|  | **head-hindwing base** |  | **0.000** |  |  |  |  |
|  | **tibia-hindwing base** |  | **0.000** |  |  |  |  |
|  | **tympanum-hindwing base** |  | **0.000** |  |  |  |  |
|  | **pronotum-hindwing base** |  | **0.000** |  |  |  |  |
|  | **sternum-hindwing base** |  | **0.000** |  |  |  |  |
|  | **tegmina-hindwing base** |  | **0.000** |  |  |  |  |
