# Supplementary material for: Host-parasite interactions between Acrididae (Orthoptera: Caelifera) and Parasitengona (Acari: Trombidiformes) in the southwestern Zagros Mountains, Iran
Source: Sci Rep. 2026 Jun 14;16:27151. doi: 10.1038/s41598-026-56417-5 (PMC13526880; doi:10.1038/s41598-026-56417-5)
Supplement: Supplementary file 2 [file 41598_2026_56417_MOESM2_ESM.docx]

| **Character** | ***C. krendowskyi***  **n=11** | ***C. baluchestanica***  **n=33** | ***C. nazaleae***  **n=16** | ***C. ahwazensis***  **n=12** | ***L*. (*L*.) *darvishi***  **n=11** | ***L*. (*L*.) *mallahae***  **n=8** | ***L*. (*L*.) *etesamiae***  **n=1** | ***A. lorestanicus***  **n=17** | ***T.hessabyi***  **n=7** | ***P. aginapacia***  **n=6** | **Character** | ***E. sorbasiensis***  **n=125** | ***E. parishanensis***  **n=3** | ***E.trigonum***  **n=25** |
| --- | --- | --- | --- | --- | --- | --- | --- | --- | --- | --- | --- | --- | --- | --- |
| L | 82--118 | 92 | 96-115 | 81-92 | 102-110 | 84-93 | 149 | - | 167-172 | 137-149 | PLN | 12-24 | 24-26 | 12-32 |
| W | 112-123 | 88 | 67-101 | 103-121 | 110-123 | 79-87 | 137 | - | 132-143 | 166-187 | PSL | 58-91 | 77 | 54-84 |
| AW | 78-90 | - | 55-84 | 77-88 | 90-94 | 65-72 | 110 | - | 95-108 | 96-101 | PSW | 89-110 | 130-134 | 106-150 |
| PW | 110-120 | - | 62-82 | 88-103 | 98-106 | 72-77 | 139 | - | 59-77 | 139-156 | MPS | 53-91 | 101 | 67-92 |
| Ta I | 178-190 | 115-132 | 190-216 | 110-128 | 152-155 | 132-156 | 216 | 117-122 | 99-121 | 192-209 | LPS | 77-118 | 118-120 | 68-112 |
| Ti I | 216-246 | 117-132 | 228-274 | 108-120 | 187-192 | 144-175 | 305 | 187-197 | 48-53 | 84-91 | lb | 7-10 | 10 | 11-13 |
| Ge I | 168-178 | 106-112 | 170-216 | 89-99 | 133-143 | 108-144 | 209 | - | 33-37 | 82-91 | 2b | 7-10 | 10-11 | 10-11 |
| Ti II | 189-202 | 107-116 | 204-254 | 96-109 | 142-160 | 120-154 | 276 | - | 44-57 | 72-74 | 3b | 7-10 | 10 | 11-12 |
| Ta III | 180-192 | 117-137 | 190-226 | 110-141 | 152-171 | 134-156 | 211 |  | 88-103 | 273-288 | Ti III | 33-40 | 53-55 | 42-48 |
| Ti III | 276-314 | 139-170 | 276-336 | 132-160 | 194-241 | 190-228 | 372 | 237-294 | 48-55 | 91-108 | - | - | - | - |

**Host-parasite interactions between Acrididae (Orthoptera: Caelifera)** **and Parasitengona (Acari: Trombidiformes) in the southwestern** **Zagros** **Mountains, Iran**

Najmeh Kiany^1^, Marjan Seiedy^1^*, Masoud Hakimitabar^2^, Mohsen Kiany^3^ and Martin Husemann^4^

Supplementary Table 2. Mite species metric data
